# Identification of essential genes by transposon insertion sequencing and genome-scale metabolic model construction in *Streptococcus suis*

**DOI:** 10.1128/spectrum.02791-24

**Published:** 2025-03-31

**Authors:** Yongqing Zhang, Ruotong Gong, Menglei Liang, Liangsheng Zhang, Xiujian Liu, Jingzi Zeng, Mengli Yan, Dexin Qiu, Rui Zhou, Qi Huang

**Affiliations:** 1 National Key Laboratory of Agricultural Microbiology, Huazhong Agriculture University 124443, Wuhan, Hubei, China; 2 College of Veterinary Medicine, Huazhong Agricultural University 627716, Wuhan, Hubei, China; 3 International Research Center for Animal Diseases, Ministry of Science and Technology of the People’s Republic of China, Wuhan, China; 4 The Cooperative Innovation Center for Sustainable Pig Production, Wuhan, China; Institute of Microbiology, Chinese Academy of Sciences, Beijing, China

**Keywords:** transposon sequencing, essential genes, genome-scale metabolic model, antimicrobial drug targets

## Abstract

Bacterial essential genes are indispensable for the survival of bacteria and therefore are attractive targets for novel anti-microbial drugs. Identifying essential genes provides a roadmap for developing novel antibiotics and anti-microbial therapies. In this study, combining high-throughput transposon sequencing (Tn-seq) and genome-scale metabolic model (GEM) construction, essential genes of *Streptococcus suis*, an important emerging zoonotic bacterial pathogen, were analyzed. A highly efficient transposon (Tn) mutagenesis system was developed in *S. suis*. This system facilitated the construction of a high-density library containing over 160,000 Tn mutants. By sequencing the library and data analysis, more than 21,000 insertion sites and 150 essential genes for growth in the rich medium were identified. Subsequently, a GEM of *S. suis* SC19 strain was constructed, and 165 essential genes were predicted via flux balance analysis (FBA). A total of 244 essential genes were obtained by combining the results of Tn-seq, and FBA performed. Gene identity analysis revealed 101 essential genes as potential anti-bacterial drug targets. Among them, apart from many known antibiotic targets, some interesting essential genes were also identified, including those involved in capsule biosynthesis, aminoacyl-tRNA biosynthesis, lipid biosynthesis, cell division, and cell signaling. This work identified essential genes of *S. suis* at the whole-genome level, providing a reference for the mining of novel anti-microbial drug targets.

**IMPORTANCE:**

Anti-microbial resistance (AMR) presents an escalating challenge, making anti-microbial drug development an urgent need. Bacterial essential genes represent promising targets for anti-microbial drugs. However, conventional approaches to identifying bacterial essential genes are time and labor intensive. Techniques such as Tn-seq and GEM construction offer a high-throughput approach for this identification. *Streptococcus sui*s is an emerging zoonotic bacterial pathogen, posing a big threat to public health as well as the pig industry, and the levels of AMR are increasing. Our study has successfully identified essential genes in *S. suis*, providing crucial insights for the discovery of new anti-microbial drug targets.

## INTRODUCTION

Anti-microbial resistance (AMR) is a challenge of global concern and is estimated to cause over 10 million deaths by 2050 if no actions are taken (1, 2). The development of antibiotics with new targets and modes of action has been proposed as a promising strategy to tackle AMR. Bacterial essential genes are indispensable for growth and survival and have higher conservation, making them promising anti-microbial drug targets (3). Identifying essential genes provides a roadmap for developing novel antibiotics and anti-microbial therapies.

Transposons (Tn’s) are important tools for functional genomic studies. Some transposases, such as *Himar1*, can insert at the thymine (T) and adenine (A) dinucleotide of DNA, therefore achieving relatively random insertions in the genome with a relatively uniform distribution of TA dinucleotides, making them very attractive tools for gene function research (4). Combined with high-throughput sequencing, transposon sequencing (Tn-seq) has been widely used to identify important genes, such as virulence factors and AMR genes (5). In the Tn-seq analysis, sequencing reads obtained from Tn mutant libraries were aligned to the genome to determine the insertion sites and frequency. Since the Tn mutants with insertions in essential genes do not survive, resulting in loss of sequencing reads, genes that are barely inserted by Tn are counted as essential genes (6). Therefore, Tn-seq provides a very efficient approach for genome-wide identification of essential genes.

Genome-scale metabolic (GEM) model construction is a powerful approach to computationally simulate the entire set of metabolic reactions within the genome of an organism, which has been widely used to analyze the cell phenotype, essential genes, antibiotic resistance, etc. (7). GEMs construction consists of four main stages: draft reconstruction, manual management, conversion to mathematical models, and network analysis (8). To accelerate GEM construction, many tools such as ModelSEED (9), Carveme (10), KBase (11), BIGG (12), and Gapseq (13) have been developed. Using flux balance analysis (FBA), *in silico* simulation of gene deletion by setting the flux value of corresponding reactions to zero in the GEM can quickly predict essential genes. The maximization of biomass production is an objective function. If FBA predicts no growth, the gene is identified as essential (7). At present, many models with multiple iterations achieved high accuracy in essential gene prediction. The *Escherichia coli* model iML1515 predicted essential genes under different conditions with a high accuracy of 93.4% (14). Besides, the *Klebsiella pneumoniae* model predicted 69 and 102 essential genes in human body fluid and sputum-macrophage conditions, respectively (15).

*Streptococcus suis* is an emerging zoonotic bacterial pathogen with increasing incidence in the human population and also one of the most important bacterial pathogens in pig farming, posing a serious threat to global public health and the pig industry (16–18). The use of antibiotics is the main strategy against *S. suis* infection. However, epidemiological studies have demonstrated that the AMR is increasing among *S. suis* isolates (19–23). Therefore, the development of antibiotics with new targets is of great significance.

So far, there have been no studies that reported the essential genes of *S. suis*. In this work, a novel efficient Tn mutagenesis system was developed for *S. suis*, and a highly saturated Tn mutant library was constructed. By sequencing the library, candidate essential genes of *S. suis* were identified. Then, a genome-scale metabolic model of the *S. suis* SC19 strain named SS-SC19-MS1 was constructed. Essential genes were predicted by using this model. By combining the results from Tn-seq and GEM analysis, a total of 244 essential genes were obtained. Homology analysis by comparing the *S. suis* essential genes with those reported in other *Streptococci* revealed potential anti-streptococcal drug targets. In summary, we have identified essential genes of *S. suis* in the rich medium by Tn-seq analysis and genome-scale metabolic network model construction, providing a reference for the mining of novel anti-microbial drug targets.

## MATERIALS AND METHODS

### Bacterial strains, growth conditions, and plasmid construction

**Fig 1:**
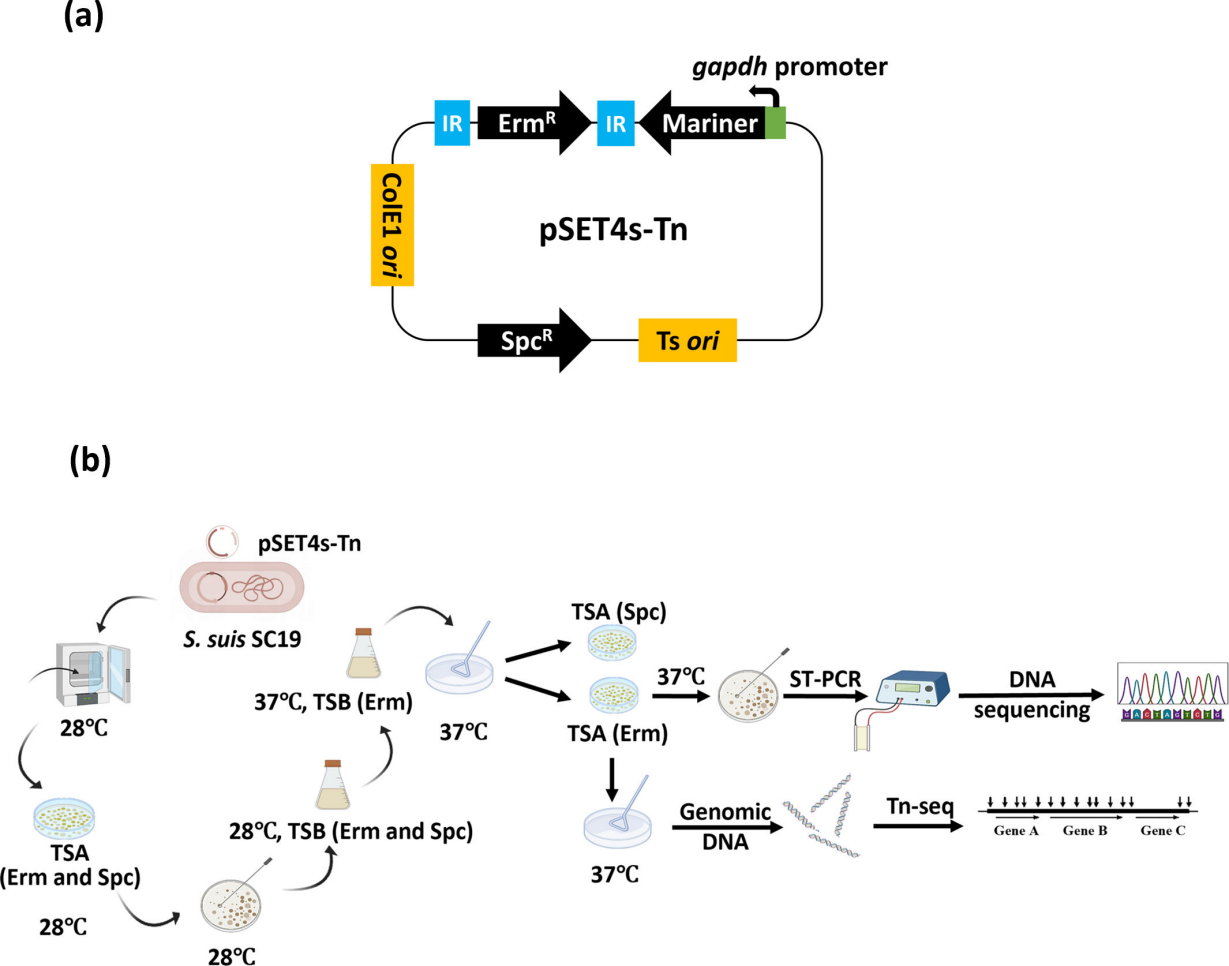
The transposon (Tn) mutagenesis system for *S. suis*. (a) The schematic diagram of pSET4s-Tn plasmid. The plasmid contains an erythromycin resistance cassette (Erm^R^) flanked by two terminal inverted repeats (IRs), a spectinomycin resistance cassette (Spc^R^), an expression cassette for the *Himar1* transposase, an *S. suis* temperature-sensitive replication element, Ts *ori*, and an *E. coli* replication element, ColE1 *ori*. (b) The procedure for highly efficient transposition in *S. suis*. The *Himar1* transposase recognized IRs and cleaved the Erm^R^ in the pSET4s-Tn plasmid of *S. suis* at 28℃, and Erm^R^ was randomly inserted into the TA sites of the *S. suis* genome. Then, the plasmid was cured from *S. suis* at 37℃, generating a transposon mutant with only erythromycin resistance. Single colonies were scraped from the plates and mixed, which constituted the Tn library.

*S. suis* SC19 strain is a clinical isolate serotype 2 strain *S. suis* SC19 that was isolated from the outbreak in Sichuan Province of China in 2005 (24). The GenBank accession number is NZ_CP020863.1 (25). *S. suis* cells were grown at 37°C with shaking at 180 rpm in tryptic soy broth (TSB) or on tryptic soy agar (TSA) (Difco, France) plates containing 5% inactivated newborn bovine serum (Sijiqing Biotech, Hangzhou, China). *E. coli* strain DH5α was used for plasmid propagation and cultured in lysogeny broth (LB) or on LB agar plates at 37°C. Erythromycin and spectinomycin were used at 5 and 100 µg/mL, respectively, when necessary (26). The plasmid pSET4s-Tn used for transposition in *S. suis* was constructed by cloning a DNA fragment containing the *Himar1* transposase cassette and the Tn into the pSET4s vector (26) between BglII and HindIII sites (Fig. 1a). The *Himar1* transposase gene was cloned from pSAM_Cam (27), and the Tn contained an erythromycin resistance cassette flanked by two terminal inverted repeats (IRs).

### Identification of insertion sites in the Tn mutant

The Tn insertion site for the individual Tn mutant was determined using semi-random two-step PCR (ST-PCR) (28). Briefly, the genomic DNA was extracted from the Tn mutant. The first-round PCR was performed using primers erm5-4 and arb2 (Table S1) with the genomic DNA as the template. The PCR product was used as the template for a second-round PCR using primers erm5-2 and arb3 (Table S1). The PCR product was then purified and sequenced. The Tn insertion site was determined by mapping the sequencing result to the *S. suis* SC19 genome using the basic local alignment search tool (BLAST). To further identify the copy numbers of Tn inserted into the genome, randomly selected Tn mutants were subjected to whole-genome sequencing using an Illumina NovaSeq platform. The sequencing reads were trimmed to remove the adapter sequences and low-quality reads. The clean reads were assembled using SPAdes (29), and GenBank accession numbers of five Tn mutants are PRJNA1168874, PRJNA1169218, PRJNA1169210, PRJNA1169204, and PRJNA1169387. The Tn insertion sites were determined by searching the Tn sequences in the assembled genome sequences.

### Construction of a transposon mutant library of *S. suis*

The pSET4s-Tn plasmid was transformed into *S. suis* by electroporation (2.5 kV, 25 mF, and 200 Ω). A single colony of the transformant was inoculated into TSB containing erythromycin and spectinomycin and cultured at 28°C for 13 hours with shaking. Then, 500 µL of the bacterial culture was transferred to 10 mL TSB containing erythromycin, followed by culture at 37°C for 12 h with shaking. The culture was then spread on TSA plates containing erythromycin and incubated at 37°C overnight. Then, 100 single colonies were randomly selected from each plate and subjected to an antibiotic resistance test. The colonies showing Erm resistance but not Spc resistance were taken as candidate Tn mutants. These mutants were further identified by PCR to detect the presence of the Tn fragment and the absence of the plasmid backbone. The efficiency of transposon insertion was determined by calculating the proportion of these Spc-resistant colonies. Finally, the colonies were scraped off from the plate, mixed, snap-frozen in liquid nitrogen, and stored at −80°C.

### Sequencing of the Tn mutant library

The library sequencing was performed as previously described (30). Briefly, the genomic DNA was extracted from the Tn mutant library. Then, the TIANSeq Fragment/Repair/Tailing Module kit (Cat # NG301; TIANGEN Biotech, China) was used for DNA fragmentation, end repair, and 3′ end dA tailing. The Illumina universal adapters were added using the TIANSeq Quick Connect Module (cat # NG303, TIANGEN Biotech), which was then purified using Magic DNA Select Beads (cat # M323; Magic Bio, China). Primer pair LTn-F/R was used in the first-round PCR amplification for the enrichment of the DNA region flanking the Tn (Table S1). The PCR product was purified and used as the template for a second-round PCR amplification using the P5/P7 primer pair (Table S1), and the amplification product was purified, which was used to construct the Illumina sequencing library. The DNA library was sequenced using an Illumina NovaSeq platform by Woosen Biotechnology (Hangzhou, China). The raw reads of Tn-seq were deposited to the National Center for Biotechnology Information (NCBI) Sequence Read Archive database under accession number PRJNA1209359.

### Tn-seq data analysis

The raw sequencing data were subjected to preliminary statistics using SeqKit software (31). Sequence screening based on primers was performed using the TPP tool in the TRANSIT package (32), and eligible sequences were aligned with genomic sequences to obtain insertion information for each locus. Essential genes were predicted using the Gumbel analysis process in the TRANSIT package (32).

### Gene homology analysis

Comparison of the *S. suis* SC19 essential gene with the eukaryotic human (*Homo sapiens*), mouse (*Mus musculus*), and pig (*Sus scrofa*) genomes was performed using BLAST on the UniProt website, and genes with identity of less than 40% were usually considered safe drug targets (33). Homology comparison of the essential genes with those reported in the core genome of *Streptococcus*, *Streptococcus pneumoniae* D39, *Streptococcus equi* subspecies *equi* 4047, *Streptococcus agalactiae* A909, and *E. coli* K-12 was performed using NCBI BLAST-2.14.0+. Annotation was performed using Kyoto Encyclopedia of Genes and Genomes (KEGG) BlastKOALA, and enrichment analysis was conducted using KEGG Mapper (34, 35). Cluster of Orthologous Groups (COG) cluster analysis was performed using eggNOG Mapper v.2 (36).

### Genome-wide metabolic model construction and FBA analysis

A metabolic network model of *S. suis* SC19 was constructed by using the ModelSEED database (9). ModelSEED is an online tool that facilitates the semi-automated construction of GEMs. The genome sequence of *S. suis* SC19 was used as the input, and the ModelSEED tool was used to predict the gene presence and their functions, relying on the annotation databases and tools. The relevant biochemical information of the genes, including their related metabolites, reactions, and pathways was used to match the predicted gene functions to known biochemical reactions in the database, generating a draft metabolic model. This model consists of metabolites connected by reactions representing the biochemical transformations that occur within the organism. This network forms the foundation of the GEM. The gap-filling function was used to add missing reactions to the model to obtain the final model. The model was then subjected to FBA using the COBRApy toolbox (37). All optimizations were performed utilizing the Gurobi solver (academic license acquisition) (38). Fluxer was used for model visualization (39).

## RESULTS

### Construction of an efficient transposon mutagenesis system in *Streptococcus suis*

To facilitate Tn mutant library construction of *S. suis*, a new Tn mutagenesis plasmid pSET4s-Tn was constructed, which contained an erythromycin resistance cassette (Erm^R^) flanked by two terminal IRs, a spectinomycin resistance cassette for plasmid selection, an expression cassette for the *Himar1* transposase, and the backbone of the temperature-sensitive shuttle plasmid pSET4s (Fig. 1a). By using this plasmid, Tn mutagenesis was achieved (Fig. 1b). The results of ST-PCR (28) analysis of 20 randomly selected Tn mutants revealed that the Erm^R^ was inserted into the T/A sites of different positions of the *S. suis* genome (Table 1). To further determine the copy number of the Tn inserted into the genome, five randomly selected Tn mutants were subjected to genome sequencing, and the Tn fragment was aligned with the whole-genome sequence to determine the insertion sites as well as the copy number of Tn. The results showed that each mutant contained a single copy of the Tn. Then, the Tn mutagenesis procedure was optimized to ensure the best efficiency of transposition and plasmid curation. After optimization, a high Tn mutagenesis efficiency of over 98% was achieved, which was calculated as the proportion of the Tn mutants among the randomly selected colonies. Therefore, a highly efficient Tn mutagenesis system in *S. suis* was successfully constructed.

**TABLE 1:** Transposon insertion sites in 20 mutants

No.	Mutant strain	Insertion site (bp)	Gene locus tag or name
1	P1-1	1,686,543	B9H01_RS08600
2	P1-3	1,603,789	Intergenic region between B9H01_RS08150 and B9H01_RS08155
3	Y1-1	868,518	B9H01_RS04405
4	P1-7	998,741	B9H01_RS05015
5	P1-9	50,372	B9H01_RS00315
6	1–6	45,902	*purB*
7	PX1-2	1,070,362	Intergenic region between B9H01_RS05395 and B9H01_RS05400
8	PX1-3	392,029	B9H01_RS02045
9	PX1-4	953,247	B9H01_RS04785
10	PX1-5	455,776	Intergenic region between B9H01_RS02375 and B9H01_RS02380
11	PX1-6	1,649,897	B9H01_RS08425
12	PX1-8	1,493,811	B9H01_RS07545
13	PX1-9	1,516,827	B9H01_RS07695
14	PX1-10	1,435,552	B9H01_RS07215
15	P3-1	794,803	B9H01_RS03995
16	P3-2	811,215	B9H01_RS03995
17	P3-3	898,171	B9H01_RS04555
18	P3-4	222,735	*treR*
19	P3-7	1,668,002	B9H01_RS08520
20	P3-10	1,357,851	*feoB*

### Transposon mutagenesis library construction and sequencing

**Fig 2:**
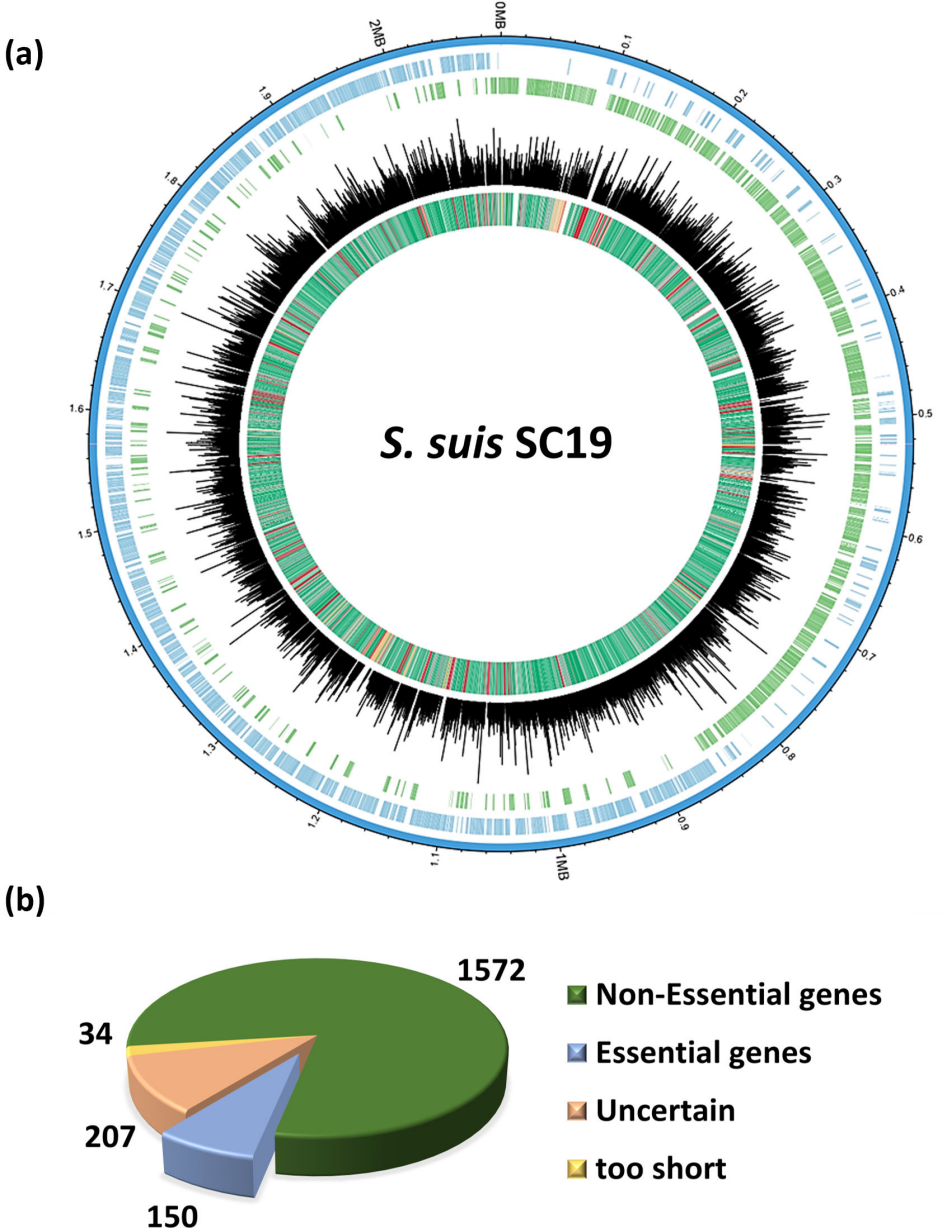
Analysis of transposon insertion sites and essential genes in the genome of *S. suis*. (a) Transposon insertion site distribution. The indications of each circle from the outside are *S. suis* CDS (+), *S. suis* CDS (−), number of reads per insertion locus, type of each gene (green for non-essential genes, blue for essential genes based on Bayesian/Gumbel method, orange for uncertain genes, yellow for too short genes). (b) Pie chart display of the result of essential gene analysis.

By using the above Tn mutagenesis system, a library containing approximately 160,500 Tn mutants was constructed. The library was subject to high-throughput sequencing as previously described (30). The results showed that a total of 21,746 insertion sites were identified, and 1,771 genes were disrupted by Tn insertion. The library saturation was 86.98% (1,771 of 2,036), with an average of 12.28 Tn insertions per disrupted gene and an average of 746.07 reads matched to each disrupted gene (Fig. 2a). These results showed that the Tn was inserted randomly and relatively uniformly into the genome of *S. suis*.

### Analysis of candidate essential genes

**Fig 3:**
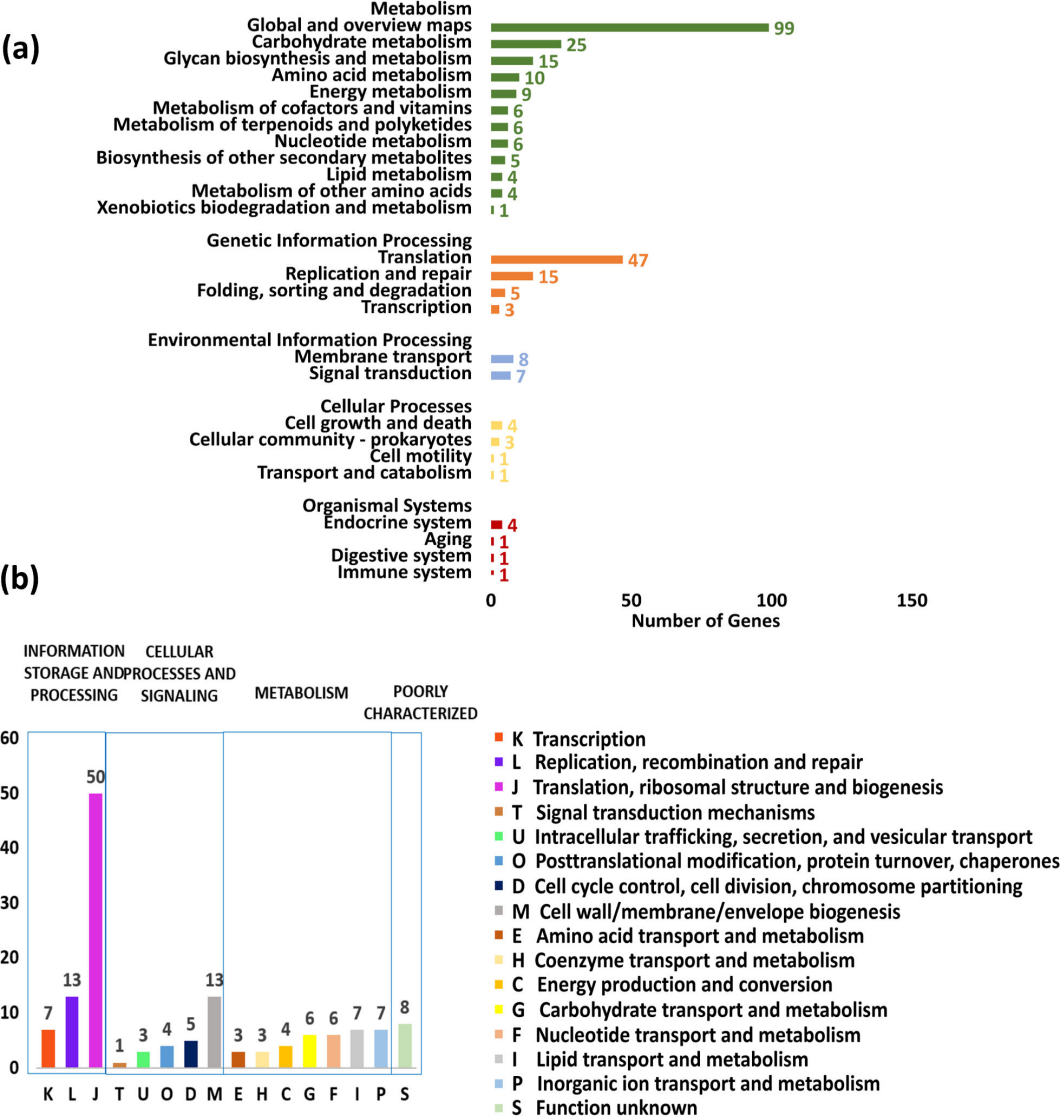
Enrichment analysis of *S. suis* SC19 essential genes obtained by Tn-seq. (a) KEGG pathway enrichment of *S. suis* SC19 essential genes. (b) COG enrichment analysis of *S. suis* SC19 essential genes. Genes will be randomly assigned to one of the subgroups if a gene is assigned to two or more COG subgroups.

By using the Gumbel analysis in the TRANSIT software package (32), essential genes and conditional genes were predicted. The results showed that 150 candidate essential genes (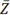
_*i*_ > 0.9966) (Table S2), 1,572 no-essential genes (0 < 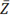
_*i*_ < 0.0454) whose posterior probability of essentiality exceeded the dynamic thresholds of essentiality, 34 short genes (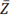
_*i*_ = −1) who were too small for reliable analysis, and 207 uncertain genes (0.9966 < 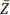
_*i*_＜0.0454) were identified (Fig. 2b). KEGG pathway enrichment of the 150 essential genes revealed that most of them were involved in metabolism, including carbohydrate metabolism, amino acid metabolism, glycan biosynthesis, and genetic information processing, including translation, replication, and repair (Fig. 3a). By COG enrichment, most of the essential genes were classified into four major categories (Fig. 3b). Forty-seven percent of the essential genes were involved in information storage and processing, including categories K (transcription), L (replication, recombination and repair), and J (translation, ribosomal structure, and biogenesis). Some genes in the cellular process and signaling, as well as metabolism, were also identified as essential (Fig. 3b).

### Construction of a genome-scale metabolic model of *Streptococcus suis* and prediction of essential genes

**Fig 4:**
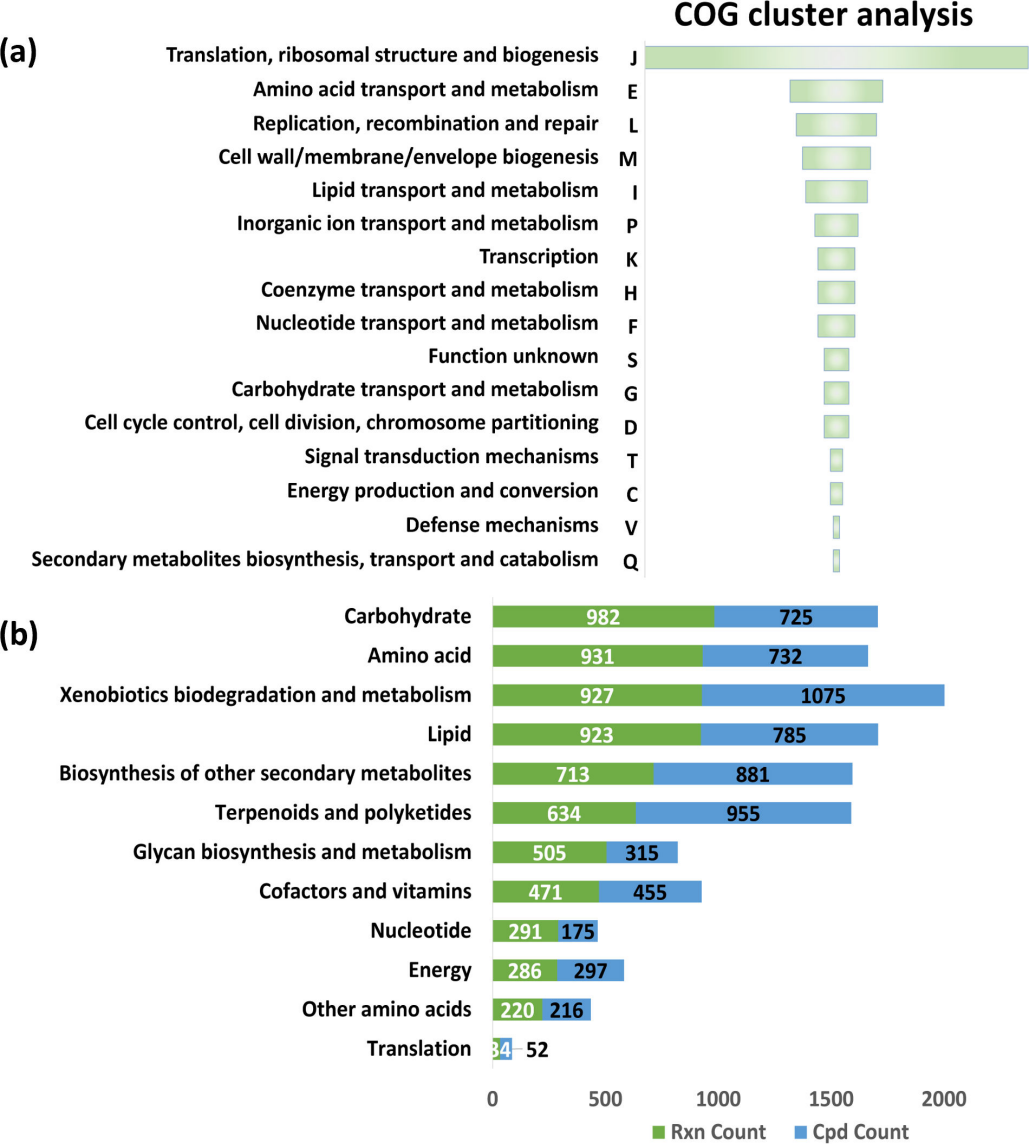
Enrichment analysis of *S. suis* SC19 essential genes predicted by the metabolic network model. (a) COG enrichment analysis. Genes will be randomly assigned to one of the subgroups if a gene is assigned to two or more COG subgroups. (b) Reactions and metabolite count involved in *S. suis*. The reaction (Rxn) counts and metabolite (Cpd) counts involved in KEGG substrates in the model. The reactions and metabolites are involved in more than one type of pathway.

Genome-scale metabolic model construction is another popular approach to predicting essential genes. Therefore, a genome-scale metabolic model of *S. suis* SC19 strain named SS-SC19-MS1 was constructed by ModelSEED. This model includes 1,089 reactions (785 reactions associated with genes), 71 exchange reactions, 482 genes out of 2,036 total genes (more than 23.7% coverage), 1,164 metabolites, and 163 pathways (https://osf.io/g8a9w/files/osfstorage). This model could perform simulations and generate biomass with a value of 24.1 g/m^2^. Subsequently, essential genes analysis was performed by FBA, which predicted 165 essential genes (Table S2). COG clustering analysis with these genes suggested that most of them were clustered to category J (translation, ribosomal structure, and biogenesis), which was consistent with the Tn-seq results. Most of the other genes fell into categories E (amino acid transport and metabolism), L (replication, recombination, and repair), M (cell wall/membrane/envelope biogenesis), and I (lipid transport and metabolism) (Fig. 4a). The reaction counts and metabolite counts involved in KEGG substrates in the model were analyzed. The results showed that most metabolic reactions in *S. suis* involved carbohydrate, amino acid, and lipid metabolism, and more than 700 metabolites were involved in these reactions (Fig. 4b).

### Analysis of potential anti-microbial drug targets

**Fig 5:**
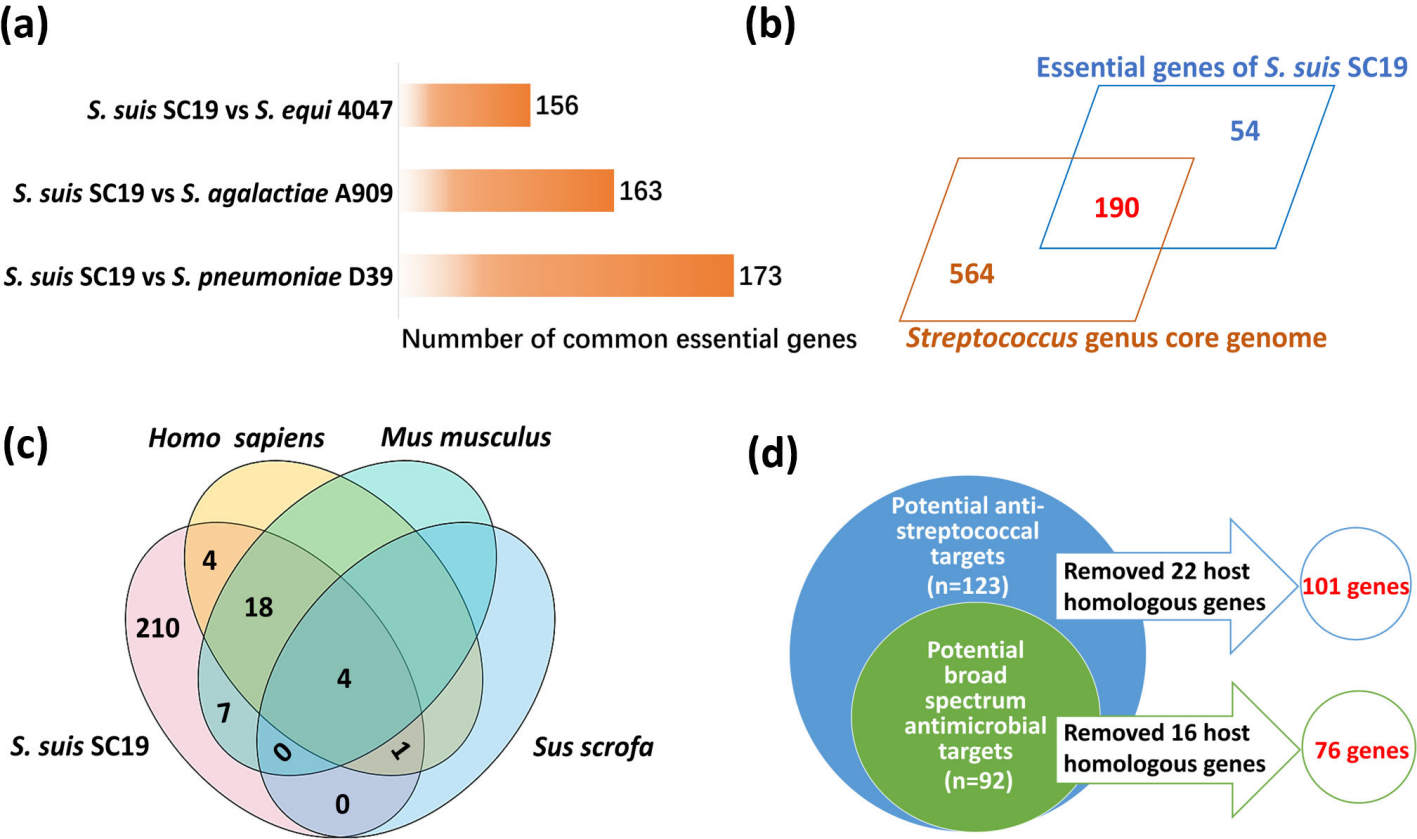
Comparison of the essential gene of *S. suis* SC19 with that of prokaryotes. (a) Comparison of the *S. suis* essential genes with those reported in other *Streptococcus* spp. The strains included *S. pneumoniae* D39 (*n* = 391), *S. equi* subspecies *equi* 4047 (*n* = 306), *S. agalactiae* A909 (*n* = 290), and *S. suis* SC19. (b) Comparison of the *S. suis* essential genes with the core genome of the *Streptococcus* genus. (c) Identity comparison between the proteins encoded by the *S. suis* essential gene and the whole proteome of *Homo sapiens*, *Mus musculus*, and *Sus scrofa*. Two hundred ten essential genes in *S. suis* might be non-conservation for the three eukaryotes. (d) Comparison of the streptococcal essential genes with those in *E. coli* K12. Essential genes sharing identity over 40% with host genes (22 genes) were removed from the potential anti-streptococcal targets (123 genes) as well as the potential broad-spectrum targets (92 genes).

By combining the results of Tn-seq and genome-scale metabolic model analysis, a total of 244 candidate essential genes of *S. suis* were obtained, which were clustered into 18 COG categories (Table S2). Most of them were classified into category J (translation, ribosomal structure, and biogenesis; 84 of 244), mainly including those encoding ribosomal proteins, enzymes involved in aminoacyl-tRNA synthesis, and several proteins involved in translation (Table S2). The second largest categories were category M (cell wall/membrane/envelope biogenesis; 20 of 244) and category L (replication, recombination, and repair; 20 of 244). The category M genes were mainly involved in the biosynthesis of cell wall structures, including peptidoglycan synthesis (*pbp2X*, *mraY*, *murB*, *murC*, *murG*, *murI*, *ddl*, and *glmS*), capsule synthesis (*galE*, *galU*, *neuB*, and *neuC*), and the biosynthesis of other cell wall molecules (*rmlA*, *rmlD*, *dltB*, and *dltD*). Category L genes mainly included the genes encoding DNA polymerase (*polA*, *dnaN*, *holA*, B9H01_RS03305, *dnaX*, and B9H01_RS09390), helicase (*dnaB*, *deaD*, and *pcrA*), DNA topoisomerase (*topA*, *gyrA*, and *gyrB*), and some DNA-binding proteins (B9H01_RS07955 and B9H01_RS08810). Twelve genes of category M were identified as essential, including those related to the uptake of potassium (B9H01_RS08480 and B9H01_RS08485) and other metal ions (B9H01_RS08790, B9H01_RS10000, B9H01_RS10005, and B9H01_RS10010). Genes encoding the key protein secretion system (*secA*, *secE*, and *yidC*) and a two-component system (TCS) (*vicK* and *vicR*) were also identified as essential. These 244 gene products were then compared with the essential proteins reported in other *Streptococci*, including *S. pneumoniae* D39 (*n* = 391) (40), *S. equi* subspecies *equi* 4047 (*n* = 306) (41), and *S. agalactiae* A909 (*n* = 290) (42) (Fig. 5a). The results revealed that 123 genes were identified as essential in all four *Streptococcus* strains (Fig. 5d). Compared with the other three *Streptococcus* essential gene lists, *S. suis* had 47 unique essential genes. Among them, 57.4% (27 of 47) encoded enzymes (hydrolases, isomerases, ligases, oxidoreductases, and transferases), and five genes (B9H01_RS08525, B9H01_RS08540, B9H01_RS07190, B9H01_RS01320, and *punC*) were transporters. The identified *S. suis* essential genes were also compared with the core genome of the *Streptococcus* genus as previously reported (43) and showed that 190 of them (77.87%) belong to the core genome (Fig. 5b; Table S3), suggesting that most of the candidate essential genes are conserved among *Streptococci*. Although essential genes are generally more conserved, previous studies in other *Streptococci* have also revealed that the essential genes do not completely belong to the core genome (44).

Potential antibiotic targets were then analyzed so that the genes sharing identity over 40% with the host genes were filtered from the 123 essential genes shared by the four *Streptococcus* strains, retaining 101 essential genes (Fig. 5c; Table S4). Among these essential genes, most were involved in the translation-ribosomal structure and biogenesis (COG category J, 48 of 101, 47.5%), including 14 encoding 50S ribosomal proteins, 7 encoding 30S ribosomal proteins, 21 involved in aminoacyl-tRNA synthesis, and 6 genes involved in the process of protein translation (Table 2). Twelve genes were involved in replication, recombination, and repair (COG category L, 12 of 101, 11.9%). They encoded the subunits of DNA polymerase subunits, DNA topoisomerase, or helicase, as well as the proteins involved in DNA replication initiation (Table 2). COG category M (cell wall/membrane/envelope biogenesis) represents the third largest group that the essential genes fell in, including seven genes involved in peptidoglycan biosynthesis and three in capsule biosynthesis (Table 2). Eight genes belong to the lipid transport and metabolism (COG category I), participating in the biosynthesis of fatty acids and terpenoids (Table 2). The remaining genes were distributed in COG category K (transcription, *n* = 4), G (carbohydrate transport and metabolism, *n* = 4), F (nucleotide transport and metabolism, *n* = 3), P (inorganic ion transport and metabolism, *n* = 2), H (coenzyme transport and metabolism, *n* = 1), C (energy production and conversion, *n* = 1), D (cell cycle control, cell division, and chromosome partitioning; *n* = 1), O (posttranslational modification, protein turnover, and chaperones; *n* = 1), T (signal transduction mechanisms, *n* = 1), and S (function unknown, *n* = 5) (Table 2). These genes were further compared with the essential genes reported in the *Escherichia coli* K-12 strain (*n* = 358) (45) (Fig. 5d; Table 2, with underline), and it was found that 72 genes remained after removing 16 essential genes with higher host identity. Most of them were involved in the translation-ribosomal structure and biogenesis (COG category J, 40 of 76, 52.6%). The second and third largest groups fell in COG category L (replication, recombination, and repair, *n* = 7) and COG category M (cell wall/membrane/envelope biogenesis, *n* = 6), respectively. These genes could be potential targets of broad-spectrum anti-microbials.

**TABLE 2:** Essential genes identified in all four *Streptococcus* spp.^*a*^

COG category	Function	Genes
Translation, ribosomal structure, and biogenesis (J)	Ribosomal proteins	*rplQ*, *rplC*, *rplW*, *rplV*, *rpsC*, *rplP*, *rpmC*, *rplN*, *rpmD*, *rplO*, *rplJ*, *rpsJ*, *rplB*, *rplF*, *rpsE*, *rpsM*, *rpsG*, *rpmI*, *rpsU*, *rpmE*, *rpsD*
	Aminoacyl-tRNA synthesis	*glyQ*, *gatC*, *gatA*, *gatB*, *valS*, *ileS*, *asnS*, *pheS*, *metG*, *thrS*, *serS*, *leuS*, *tyrS*, *hisS*, *fmt*, *pheT*, *alaS*, *glyS*, *cysS*, *gltX*, *argS*
	Others	*ccA*, *infC*, *prfB*, *tsf*, *rnpA*, *tilS*
Replication, recombination, and repair (L)	DNA polymerase	*dnaN*, *dnaX*, *holA*
	DNA topoisomerase	*gyrA*, *gyrB*
	Helicase	*deaD*, *dnaB*
	Initiation	*dnaA*, *dnaI*, *dnaG*, B9H01_RS08340
Cell wall/membrane/envelope biogenesis (M)	Peptidoglycan biosynthesis	*murG*, glmS*, *ddl*, *murI*, *murC*, *mraY*, *pbp2X*
	Capsule biosynthesis	rmlA*, rmlD*, galU*
Lipid transport and metabolism (I)	Fatty acid biosynthesis	*fabF*, *fabD*, *fabH*, *acpP*
	Terpenoid biosynthesis	*mvaA*, *mvaD*, *mvaS*, *uppS*
Transcription (K)	RNA polymerase	*rpoD*, *rpoA*, *rpoB*, *rpoC*
Carbohydrate transport and metabolism (G)	PTS system	*ptsP*, *ptsH*
	NADP biosynthesis	*nadK*
	Glycolysis	*pyk*
Nucleotide transport and metabolism (F)	Nucleotide biosynthesis	*tmk*, *nrdE*, *pyrH*
Inorganic ion transport and metabolism (P)	Metal ion transporter	B9H01_RS10010, B9H01_RS08790
Coenzyme transport and metabolism (H)	FAD synthetase	*ribC*
Energy production and conversion (C)	ATP synthase subunit	*atpG*
Cell cycle control, cell division, and chromosome partitioning (D)	Cell division	*ftsZ*
Posttranslational modification, protein turnover, and chaperones (O)	tRNA modification	*tsaD*
Signal transduction mechanisms (T)	Response regulator	*vicR*
Function unknown (S)	Unknown	*rnz*, B9H01_RS06415, *cdaA*, B9H01_RS08950, B9H01_RS09435

^*a*^ The essential genes that were also reported to be essential in *E. coli* K-12 were underscored. * indicates genes that may be allocated to different pathways depending on the functional enrichment method.

## DISCUSSION

Identifying essential genes is an important approach for drug target discovery and antibiotic development. In this study, by utilizing Tn-seq and genome-scale metabolic model construction, a total of 244 candidate essential genes were identified in the *S. suis* SC19 strain. By comparing these essential genes with those reported in other *Streptococci* and *E. coli*, potential drug targets were analyzed. Our study is the first report to identify essential genes in *S. suis* and will be valuable for novel antibiotic development.

The establishment of an efficient Tn mutagenesis system is one of the highlights of this study. We have previously utilized a Tn mutagenesis system with Tn917 transposon to generate mutants in *S. suis* (46). However, the efficiency was not high enough to easily construct highly saturated libraries. Moreover, the Tn917 transposon has been reported to have biases toward the replication terminus (47). Arenas et al. reported the use of *Himar1* transposon in the construction of a mutant library of *S. suis* (48). They used an *in vitro* approach to assemble Tn complex in which genomic DNA was incubated with the transposon and the transposase followed by transformation with *S. suis*. In this study, we used the temperature-sensitive plasmid pSET4s-Tn as the backbone, which could be easily cured by temperature shift, and used the *Himar1* transposase with little insertion bias. To generate a highly saturated Tn mutant library, the key points remained as high transposition efficiency as well as successful plasmid curation. We optimized the transposition conditions, including the duration of transposition, temperature for plasmid curation, as well as the antibiotic concentrations used for mutant selection (Fig. 1a). By using this efficient Tn tool, we constructed a high-density Tn library (containing over 160,000 transposon mutants) which was much larger than the one constructed by Arenas et al. (48), increasing the accuracy of the essential gene list. However, it should be noted that a relatively long-term incubation time (13 hours) was used to ensure the curation of the temperature-sensitive plasmid used for transposition. Therefore, some slow-growing mutants would likely be eliminated by competition from faster growers within the mixed population. This bias toward excluding mutations that lead to slow growth could be severe with the Tn mutagenesis system in the current work.

The genome-scale metabolic model construction provides another precise and efficient way for the prediction of bacterial essential genes. For example, the metabolic model of *E. coli* has been continuously updated in recent years, and using the metabolic model of the iML1515 strain to predict essential genes under different conditions can achieve an accuracy of 93.4% (14). To further identify essential genes, a genome-scale metabolic model of the *S. suis* SC19 strain was constructed for the first time in this study. The model was constructed using the ModelSEED. The model contains 482 genes associated with 785 reactions, 1,164 metabolites, and 163 pathways, which harbor comparable genes and pathways with a reported model of *Streptococcus pyogenes* (49). This model could successfully perform simulations and generate biomass and therefore was used for essential gene prediction. It is worth noting that the quality of the model determines the accuracy in predicting gene essentiality. Lieven et al. developed a test suite named MEMOTE to assess GEM quality, and higher MEMOTE scores indicated better GEM quality (50). Hirose et al. constructed a high-quality GEM of *S. pyogenes*, therefore increasing the accuracy of gene essentiality predictions from 73.6% to 92.6% (51). Therefore, further optimization of the model will be needed.

Although Tn-seq analysis and GEM construction provide a powerful approach to predicting essential genes, they have limitations. Insertion of transposons near the termination codon end of genes generally does not result in loss of function, causing some essential genes to be classified as non-essential genes (52). Also, due to the polar effects of Tn insertion, disruptions in essential genes may lead to misidentification, where downstream non-essential genes cotranscribed with them are erroneously categorized as essential (53). In terms of using GEM to predict essential genes, it is mainly focused on important metabolic pathways but may overlook regulatory mechanisms or transport systems. For example, the two-component system VicK/VicR is a well-known essential regulatory system in *Streptococcus* (54). It was revealed as essential in the Tn-seq analysis but was not predicted as essential in the GEM analysis. Similarly, 79 essential genes identified by Tn-seq were classified as non-essential. This could have resulted from the size or saturation of the Tn library, or it may be the result of using a non-condition-specific metabolic model for prediction. In summary, Tn-seq screening of essential genes requires a large mutant library, while GEM prediction of essential genes requires a fully annotated bacterial genome with sufficient mapping to the metabolic database. Further validation of these essential genes using other methods, such as clustered regularly interspaced short palindromic repeats interference, will be very valuable (55).

To analyze potential anti-microbial drug targets, a total of 244 candidate essential genes were analyzed by combining the results of the Tn-seq and GEM prediction. These genes were compared with those reported in other *Streptococci*, including *S. pneumoniae* D39, *S. equi* subspecies *equi* 4047, and *S. agalactiae* A909, and 101 genes were obtained to be present in all the strains but with no homology to the host. We found that the proteins encoded by these genes include existing antibiotic targets, such as ribosomal subunit proteins (macrolides), peptidoglycan synthases (beta-lactams), DNA topoisomerases (quinolones), and RNA polymerases (rifampicin) (56). However, some new potential anti-bacterial drug targets have also been discovered, such as the proteins involved in capsule synthesis (RmlA, RmlD, and GalU), aminoacyl tRNA synthetases (GlyQ, GatC, ValS, etc.), lipid synthesis-related proteins (FabF, FabD, and FabH), cell division proteins (FtsZ), and two-component system proteins (VicR). These results provide useful information to the field of drug development and design.

Extensive efforts have been taken to develop inhibitors against these non-canonical antibiotic targets. FabF inhibitor platensimycin (57) and FabH inhibitor platencin (58), amycomycin (59), and 1,3,5-oxadiazin-2-ones named Oxa1 and Oxa2 (60) have been reported to be effective against *Staphylococcus aureus* (61). FtsZ is an essential cytoskeletal protein conserved among bacteria and is deemed a promising anti-microbial drug target (62). Several FtsZ inhibitors have been developed, including PC190723 and TXA709, showing very remarkable anti-microbial activity (63). Due to their important role in the translation of the genetic code, aminoacyl-tRNA synthetases have been recognized as suitable targets for the development of small molecule anti-infectives. Buckner et al. further optimized the MetRS inhibitor REP8839, and the best compounds 1717 and 2144 were shown to have broad-spectrum anti-bacterial activities against gram-positive pathogens *Staphylococcus*, *Enterococcus*, and *Streptococcus* strains (64). TCSs have long been regarded as promising anti-microbial drug targets and they are particularly attractive in *Streptococcus*, since some TCSs, such as VicR/K, have been found to be essential (65). Several compounds have been screened by *in vitro* and *in silico* screens, which showed inhibition against *S. aureus* and *Staphylococcus epidermidis* (66).

## SUPPLEMENTAL MATERIAL

Tables S1 to S4 spectrum.02791-24-s0001.pdf Primers and essential genes of the manuscript.

ASM does not own the copyrights to Supplemental Material that may be linked to, or accessed through, an article. The authors have granted ASM a non-exclusive, world-wide license to publish the Supplemental Material files. Please contact the corresponding author directly for reuse.
