# Supplementary material for: Identification of essential genes by transposon insertion sequencing and genome-scale metabolic model construction in Streptococcus suis
Source: Microbiol Spectr. 2025 Mar 31;13(5):e02791-24. doi: 10.1128/spectrum.02791-24 (PMC12053914; doi:10.1128/spectrum.02791-24)
Supplement: Tables S1 to S4 — Primers and essential genes of the manuscript. [file spectrum.02791-24-s0001.pdf]

Table S1 Nucleotide sequence primers in study

Primer	Sequence (5’-3’)	Function
Erm5-4	GGTTGAGTACTTTTTCACTCG	ST-PCR (PCR1)
arb-2	GGCCACGCGTCGACTAGTCANNNNNNNNNNGATCA	
Erm5-2	GGAATACCAACATGACGAATC	ST-PCR (PCR2)
arb-3	GGCCACGCGTCGACTAGTCA	
Erm-R	GACGATATTCTCGATTGACC	Plasmid resistance or component identification
Erm-F	GAAACCGATACCGTTTACG	
Spc-F	GATCAGGAGTTGAGAGTG	
Spc-R	CTCTTCTCACATCAGAAAATGG	
Ori-F	GTAATCTGCTGCTTGCAAAC	
Ori-R	CGCTGTAGGTATCTCAGTTC	
P5	AATGATACGGCGACCACCGAGATCACACTCTTTCCCTACACGACGCTCTTCC	Transposon insertion sequencing (Tn-seq)
P7	CAAGCAGAAGACGGCATACGAAGCGCCTACGAGGAATTTGTATCG	
LTn-F	ACACTCTTTCCCTACACGACGCTCTTCCGATCT	
SS-LTn-R	GTGACTGGAGTTCAGACGTGTGCTCTTCCGATCTCACTATCAACACACTCTT	
Tn-F	ATGTTGCGATTAATAGATCTGTCGACCTGCAGTAAG	Plasmid construction
Tn-R	ACCATGATTACGCCAAGCTTTAACAGGTTGGATGATAAGTCC	

Table S2 Essential genes from Tn-seq screening and metabolic model prediction

No.	***S. suis*** **SC19 Locus tag**	**Name**	**Description**	**COG category**	**Metabolic model predicting essential genes**	**Tn-seq screening essential genes**
1	B9H01_RS00005	*dnaA*	chromosomal replication initiator protein DnaA	L	Yes	Yes
2	B9H01_RS00010	*dnaN*	DNA polymerase III subunit beta	L	Yes	No
3	B9H01_RS00035	-	aminoacyl-tRNA hydrolase	J	No	Yes
4	B9H01_RS00065	*tilS*	tRNA lysidine(34) synthetase TilS	D	Yes	No
5	B9H01_RS00205	-	ribose-phosphate diphosphokinase	EF	No	Yes
6	B9H01_RS00440	*rpsJ*	30S ribosomal protein S10	J	Yes	Yes
7	B9H01_RS00445	*rplC*	50S ribosomal protein L3	J	Yes	No
8	B9H01_RS00450	*rplD*	50S ribosomal protein L4	J	Yes	No
9	B9H01_RS00455	*rplW*	50S ribosomal protein L23	J	Yes	Yes
10	B9H01_RS00460	*rplB*	50S ribosomal protein L2	J	Yes	No
11	B9H01_RS00465	*rpsS*	30S ribosomal protein S19	J	Yes	No
12	B9H01_RS00470	*rplV*	50S ribosomal protein L22	J	Yes	Yes
13	B9H01_RS00475	*rpsC*	30S ribosomal protein S3	J	Yes	Yes
14	B9H01_RS00480	*rplP*	50S ribosomal protein L16	J	Yes	Yes
15	B9H01_RS00485	*rpmC*	50S ribosomal protein L29	J	Yes	No
16	B9H01_RS00490	*rpsQ*	30S ribosomal protein S17	J	Yes	Yes
17	B9H01_RS00495	*rplN*	50S ribosomal protein L14	J	Yes	Yes
18	B9H01_RS00500	*rplX*	50S ribosomal protein L24	J	Yes	Yes
19	B9H01_RS00505	*rplE*	50S ribosomal protein L5	J	Yes	Yes

No.	*S. suis* SC19 Locus tag	Name	Description	COG category	Metabolic model predicting essential genes	Tn-seq screening essential genes
20	B9H01_RS00510	-	type Z 30S ribosomal protein S14	J	No	Yes
21	B9H01_RS00520	*rpsH*	30S ribosomal protein S8	J	Yes	No
22	B9H01_RS00530	*rplF*	50S ribosomal protein L6	J	Yes	Yes
23	B9H01_RS00535	*rplR*	50S ribosomal protein L18	J	Yes	Yes
24	B9H01_RS00540	*rpsE*	30S ribosomal protein S5	J	Yes	Yes
25	B9H01_RS00545	*rpmD*	50S ribosomal protein L30	J	Yes	Yes
26	B9H01_RS00550	*rplO*	50S ribosomal protein L15	J	Yes	Yes
27	B9H01_RS00560	-	adenylate kinase	F	Yes	No
28	B9H01_RS00570	*rpmJ*	50S ribosomal protein L36	J	Yes	No
29	B9H01_RS00575	*rpsM*	30S ribosomal protein S13	J	Yes	No
30	B9H01_RS00580	*rpsK*	30S ribosomal protein S11	J	Yes	Yes
31	B9H01_RS00585	*rpoA*	DNA-directed RNA polymerase subunit alpha	K	Yes	No
32	B9H01_RS00590	*rplQ*	50S ribosomal protein L17	J	Yes	Yes
33	B9H01_RS00715	-	hypothetical protein	NA	No	Yes
34	B9H01_RS00770	*tyrS*	tyrosine--tRNA ligase	J	Yes	No
35	B9H01_RS00780	*rpoB*	DNA-directed RNA polymerase subunit beta	K	Yes	Yes
36	B9H01_RS00785	*rpoC*	DNA-directed RNA polymerase subunit beta	K	Yes	Yes
37	B9H01_RS00900	*groL*	chaperonin GroEL	O	No	Yes
38	B9H01_RS00905	*rpsL*	30S ribosomal protein S12	J	Yes	Yes

No.	*S. suis* SC19 Locus tag	Name	Description	COG category	Metabolic model predicting essential genes	Tn-seq screening essential genes
39	B9H01_RS00910	*rpsG*	30S ribosomal protein S7	J	Yes	Yes
40	B9H01_RS00915	*fusA*	elongation factor G	J	No	Yes
41	B9H01_RS00925	*gap*	type I glyceraldehyde-3-phosphate dehydrogenase	G	No	Yes
42	B9H01_RS00970	*tsaD*	tRNA (adenosine(37)-N6)-threonylcarbamoyltransferase complex transferase subunit TsaD	O	No	Yes
43	B9H01_RS01245	*treC*	alpha,alpha-phosphotrehalase	G	No	Yes
44	B9H01_RS01280	*trxA*	thioredoxin	O	No	Yes
45	B9H01_RS01320	-	mechanosensitive ion channel family protein	M	Yes	No
46	B9H01_RS01335	*gdhA*	NADP-specific glutamate dehydrogenase	E	Yes	No
47	B9H01_RS01405	-	formate/nitrite transporter family protein	P	Yes	No
48	B9H01_RS01450	*rpmF*	50S ribosomal protein L32	J	Yes	No
49	B9H01_RS01455	*hisS*	histidine--tRNA ligase	J	Yes	Yes
50	B9H01_RS01505	*mvk*	mevalonate kinase	I	No	Yes
51	B9H01_RS01510	*mvaD*	diphosphomevalonate decarboxylase	I	Yes	Yes
52	B9H01_RS01515	-	phosphomevalonate kinase	I	Yes	No

No.	*S. suis* SC19 Locus tag	Name	Description	COG category	Metabolic model predicting essential genes	Tn-seq screening essential genes
54	B9H01_RS01555	*grpE*	nucleotide exchange factor GrpE	NA	No	Yes
55	B9H01_RS01730	*rpmB*	50S ribosomal protein L28	J	Yes	No
56	B9H01_RS01810	*rplS*	50S ribosomal protein L19	J	Yes	No
57	B9H01_RS01820	*gatC*	Asp-tRNA(Asn)/Glu-tRNA(Gln) amidotransferase subunit GatC	J	Yes	Yes
58	B9H01_RS01825	*gatA*	Asp-tRNA(Asn)/Glu-tRNA(Gln) amidotransferase subunit GatA	J	Yes	No
59	B9H01_RS01830	*gatB*	Asp-tRNA(Asn)/Glu-tRNA(Gln) amidotransferase subunit GatB	J	Yes	No
60	B9H01_RS01930	-	nucleotidyltransferase	S	No	Yes
61	B9H01_RS02070	*gpsB*	cell division regulator GpsB	D	No	Yes
62	B9H01_RS02100	*gmk*	guanylate kinase	F	Yes	Yes
63	B9H01_RS02105	-	DNA-directed RNA polymerase subunit omega	K	No	Yes
64	B9H01_RS02110	-	primosomal protein N'	L	Yes	No
65	B9H01_RS02115	*fmt*	methionyl-tRNA formyltransferase	J	Yes	No
66	B9H01_RS02330	*valS*	valine--tRNA ligase	J	Yes	Yes
67	B9H01_RS02365	*murG*	aspartate--ammonia ligase	E	Yes	No
68	B9H01_RS02420	*murG*	UDP-N-acetylglucosamine--N-acetylmuramyl- (pentapeptide) pyrophosphoryl-undecaprenol N-acetylglucosamine transferase	M	Yes	No

No.	*S. suis* SC19 Locus tag	Name	Description	COG category	Metabolic model predicting essential genes	Tn-seq screening essential genes
69	B9H01_RS02435	*ftsZ*	cell division protein FtsZ	D	No	Yes
70	B9H01_RS02470	*ileS*	isoleucine--tRNA ligase	J	Yes	Yes
71	B9H01_RS02620	*lysS*	lysine--tRNA ligase	J	Yes	No
72	B9H01_RS02660	-	FtsW/RodA/SpoVE family cell cycle protein	D	No	Yes
73	B9H01_RS02740	-	6-phosphofructokinase	F	No	Yes
74	B9H01_RS02745	*pyk*	pyruvate kinase	G	No	Yes
75	B9H01_RS02770	*glmS*	glutamine--fructose-6-phosphate transaminase (isomerizing)	M	No	Yes
76	B9H01_RS02805	*proB*	glutamate 5-kinase	E	Yes	No
77	B9H01_RS02810	-	glutamate-5-semialdehyde dehydrogenase	E	Yes	No
78	B9H01_RS02815	*proC*	pyrroline-5-carboxylate reductase	E	Yes	No
79	B9H01_RS02925	*neuB*	N-acetylneuraminate synthase	M	No	Yes
80	B9H01_RS02930	*neuC*	UDP-N-acetylglucosamine 2-epimerase (hydrolyzing)	M	Yes	Yes
81	B9H01_RS02940	-	GDSL-type esterase/lipase family protein	M	No	Yes
82	B9H01_RS03070	*asnS*	asparagine--tRNA ligase	J	No	Yes
83	B9H01_RS03145	-	YfcC family protein	S	Yes	No
84	B9H01_RS03200	*dltA*	D-alanine--poly(phosphoribitol) ligase subunit DltA	Q	Yes	No

No.	*S. suis* SC19 Locus tag	Name	Description	COG category	Metabolic model predicting essential genes	Tn-seq screening essential genes
85	B9H01_RS03205	*dltB*	D-alanyl-lipoteichoic acid biosynthesis protein DltB	M	Yes	No
86	B9H01_RS03215	*dltD*	D-alanyl-lipoteichoic acid biosynthesis protein DltD	M	Yes	No
87	B9H01_RS03265	-	1-acyl-sn-glycerol-3-phosphate acyltransferase	I	No	Yes
88	B9H01_RS03300	*tmk*	dTMP kinase	F	Yes	No
89	B9H01_RS03305	-	DNA polymerase III subunit delta'	L	Yes	Yes
90	B9H01_RS03430	-	ECF transporter S component	S	No	Yes
91	B9H01_RS03440	-	phosphopantothenate--cysteine ligase	H	No	Yes
92	B9H01_RS03615	*pcrA*	DNA helicase PcrA	L	Yes	No
93	B9H01_RS03745	*parE*	DNA topoisomerase IV subunit B	L	No	Yes
94	B9H01_RS03780	-	branched-chain amino acid aminotransferase	E	Yes	No
95	B9H01_RS03840	*rplU*	50S ribosomal protein L21	J	Yes	No
96	B9H01_RS03845	*rpmA*	50S ribosomal protein L27	J	Yes	Yes
97	B9H01_RS03885	-	aspartate carbamoyltransferase catalytic subunit	F	Yes	No
98	B9H01_RS03915	*rpsP*	30S ribosomal protein S16	J	Yes	Yes
99	B9H01_RS03920	-	KH domain-containing protein	S	No	Yes

No.	*S. suis* SC19 Locus tag	Name	Description	COG category	Metabolic model predicting essential genes	Tn-seq screening essential genes
100	B9H01_RS03950	*murB*	UDP-N-acetylmuramate dehydrogenase	M	Yes	Yes
101	B9H01_RS04060	*ccA*	CCA tRNA nucleotidyltransferase	J	No	Yes
102	B9H01_RS04090	-	thymidylate synthase	F	Yes	Yes
103	B9H01_RS04110	-	ribosome biogenesis GTP-binding protein YihA/YsxC	D	No	Yes
104	B9H01_RS04150	*prfA*	peptide chain release factor 1	J	No	Yes
105	B9H01_RS04770	-	transcriptional regulator	K	No	Yes
106	B9H01_RS04855	*rplJ*	50S ribosomal protein L10	J	Yes	No
107	B9H01_RS04860	*topA*	type I DNA topoisomerase	L	Yes	Yes
108	B9H01_RS04910	*ylqF*	ribosome biogenesis GTPase YlqF	S	No	Yes
109	B9H01_RS05190	*nadK*	NAD kinase	G	Yes	Yes
110	B9H01_RS05245	*gyrA*	DNA gyrase subunit A	L	Yes	Yes
111	B9H01_RS05285	*deoC*	deoxyribose-phosphate aldolase	F	Yes	No
112	B9H01_RS05300	-	type I pantothenate kinase	F	No	Yes
113	B9H01_RS05305	-	30S ribosomal protein S20	J	Yes	Yes
114	B9H01_RS05375	*pstS*	phosphate ABC transporter substrate-binding protein PstS family protein	P	No	Yes
115	B9H01_RS05405	*ribC*	bifunctional riboflavin kinase/FAD synthetase	H	Yes	Yes

No.	*S. suis* SC19 Locus tag	Name	Description	COG category	Metabolic model predicting essential genes	Tn-seq screening essential genes
116	B9H01_RS05585	*pheT*	phenylalanine--tRNA ligase subunit beta	J	Yes	Yes
117	B9H01_RS05595	*pheS*	phenylalanine--tRNA ligase subunit alpha	J	Yes	Yes
118	B9H01_RS05685	*atpG*	F0F1 ATP synthase subunit gamma	C	No	Yes
119	B9H01_RS05690	-	F0F1 ATP synthase subunit alpha	C	No	Yes
120	B9H01_RS05710	-	F0F1 ATP synthase subunit C	C	No	Yes
121	B9H01_RS05765	*smc*	chromosome segregation protein SMC	D	Yes	No
122	B9H01_RS05770	*rnc*	ribonuclease III	K	Yes	No
123	B9H01_RS05800	*ptsP*	phosphoenolpyruvate--protein phosphotransferase	G	Yes	No
124	B9H01_RS05805	*ptsH*	phosphocarrier protein HPr	G	No	Yes
125	B9H01_RS05825	*nrdH*	glutaredoxin-like protein NrdH	O	No	Yes
126	B9H01_RS05830	*nrdE*	class 1b ribonucleoside-diphosphate reductase subunit alpha	F	No	Yes
127	B9H01_RS05835	-	hypothetical protein	NA	No	Yes
128	B9H01_RS05960	-	helix-turn-helix transcriptional regulator	K	No	Yes
129	B9H01_RS05985	*alaS*	alanine--tRNA ligase	J	Yes	Yes
130	B9H01_RS06015	*pepF*	oligoendopeptidase F	E	No	Yes
131	B9H01_RS06025	*metG*	methionine--tRNA ligase	J	Yes	Yes

No.	*S. suis* SC19 Locus tag	Name	Description	COG category	Metabolic model predicting essential genes	Tn-seq screening essential genes
132	B9H01_RS06135	*rplT*	50S ribosomal protein L20	J	Yes	No
133	B9H01_RS06140	*rpmI*	50S ribosomal protein L35	J	Yes	No
134	B9H01_RS06145	*infC*	translation initiation factor IF-3	J	No	Yes
135	B9H01_RS06260	*rmlD*	dTDP-4-dehydrorhamnose reductase	M	No	Yes
136	B9H01_RS06280	*rmlA*	glucose-1-phosphate thymidylyltransferase RfbA	M	No	Yes
137	B9H01_RS06345	*rnz*	ribonuclease Z	S	No	Yes
138	B9H01_RS06410	*pyrH*	UMP kinase	F	No	Yes
139	B9H01_RS06415	-	polysaccharide biosynthesis protein	S	No	Yes
140	B9H01_RS06430	*rplA*	50S ribosomal protein L1	J	Yes	Yes
141	B9H01_RS06435	*rplK*	50S ribosomal protein L11	J	No	Yes
142	B9H01_RS06530	*ddl*	D-alanine--D-alanine ligase	M	No	Yes
143	B9H01_RS06560	*vicK*	cell wall metabolism sensor histidine kinase VicK	T	Yes	No
144	B9H01_RS06565	*vicR*	response regulator transcription factor	T	Yes	Yes
145	B9H01_RS06590	*thrS*	threonine--tRNA ligase	J	Yes	Yes
146	B9H01_RS06650	*polA*	DNA polymerase I	L	No	Yes
147	B9H01_RS06745	-	diacylglycerol kinase family protein	M	Yes	No
148	B9H01_RS06815	-	ABC transporter permease	D	No	Yes
149	B9H01_RS06825	*prfB*	peptide chain release factor 2	J	No	Yes

No.	*S. suis* SC19 Locus tag	Name	Description	COG category	Metabolic model predicting essential genes	Tn-seq screening essential genes
150	B9H01_RS06905	*rpoD*	RNA polymerase sigma factor RpoD	K	Yes	Yes
151	B9H01_RS06910	*dnaG*	DNA primase	L	No	Yes
152	B9H01_RS06920	*galE*	UDP-glucose 4-epimerase GalE	M	Yes	Yes
153	B9H01_RS06925	*rpsU*	30S ribosomal protein S21	J	Yes	Yes
154	B9H01_RS06955	-	VanZ family protein	NA	No	Yes
155	B9H01_RS06975	-	arsenate reductase	P	No	Yes
156	B9H01_RS06980	-	phosphopentomutase	G	Yes	No
157	B9H01_RS07095	*cdaA*	TIGR00159 family protein	S	No	Yes
158	B9H01_RS07190	-	beta-glucoside-specific PTS transporter subunit IIABC	G	Yes	No
159	B9H01_RS07285	*gyrB*	DNA topoisomerase (ATP-hydrolyzing) subunit B	L	Yes	Yes
160	B9H01_RS07390	*rpmE*	type B 50S ribosomal protein L31	J	Yes	Yes
161	B9H01_RS07440	*holA*	DNA polymerase III subunit delta	L	Yes	Yes
162	B9H01_RS07535	-	cystathionine gamma-synthase	E	Yes	No
163	B9H01_RS07835	-	methionine adenosyltransferase	H	Yes	Yes
164	B9H01_RS07865	*dnaX*	DNA polymerase III subunit gamma/tau	L	Yes	No
165	B9H01_RS07920	-	acetyl-CoA C-acetyltransferase	I	Yes	Yes
166	B9H01_RS07925	*mvaS*	hydroxymethylglutaryl-CoA synthase	I	Yes	Yes

No.	*S. suis* SC19 Locus tag	Name	Description	COG category	Metabolic model predicting essential genes	Tn-seq screening essential genes
167	B9H01_RS07930	*mvaA*	hydroxymethylglutaryl-CoA reductase,degradative	I	Yes	No
168	B9H01_RS07955	-	HU family DNA-binding protein	L	Yes	Yes
169	B9H01_RS07990	-	farnesyl diphosphate synthase	H	Yes	No
170	B9H01_RS08075	-	nicotinate phosphoribosyltransferase	H	Yes	No
171	B9H01_RS08180	-	ECF transporter S component	S	Yes	No
172	B9H01_RS08205	*scpB*	SMC-Scp complex subunit ScpB	D	Yes	No
173	B9H01_RS08210	-	segregation/condensation protein A	D	Yes	No
174	B9H01_RS08235	*murI*	glutamate racemase	M	Yes	No
175	B9H01_RS08245	-	diaminopimelate decarboxylase	E	Yes	No
176	B9H01_RS08275	*yidC*	membrane protein insertase YidC	U	No	Yes
177	B9H01_RS08295	*murC*	UDP-N-acetylmuramate--L-alanine ligase	M	No	Yes
178	B9H01_RS08335	*dnaI*	primosomal protein DnaI	L	Yes	No
179	B9H01_RS08340	-	replication initiation/membrane attachment protein	L	No	Yes
180	B9H01_RS08360	*gndA*	NADP-dependent phosphogluconate dehydrogenase	G	No	Yes
181	B9H01_RS08380	*deaD*	DEAD/DEAH box helicase	L	No	Yes
182	B9H01_RS08390	*mraY*	phospho-N-acetylmuramoyl-pentapeptide-transferase	M	Yes	Yes

No.	*S. suis* SC19 Locus tag	Name	Description	COG category	Metabolic model predicting essential genes	Tn-seq screening essential genes
183	B9H01_RS08395	*pbp2X*	penicillin-binding protein PBP2X	M	No	Yes
184	B9H01_RS08460	-	polyprenyl synthetase family protein	H	Yes	No
185	B9H01_RS08470	-	Gx transporter family protein	S	Yes	No
186	B9H01_RS08480	-	potassium transporter Trk	P	No	Yes
187	B9H01_RS08485	-	TrkA family potassium uptake protein	P	No	Yes
188	B9H01_RS08505	*glyS*	glycine--tRNA ligase subunit beta	J	Yes	No
189	B9H01_RS08510	*glyQ*	glycine--tRNA ligase subunit alpha	J	Yes	Yes
190	B9H01_RS08525	-	methionine ABC transporter permease	P	Yes	No
191	B9H01_RS08540	-	MetQ/NlpA family ABC transporter substrate-binding protein	P	Yes	No
192	B9H01_RS08555	*metF*	methylenetetrahydrofolate reductase [NAD(P)H]	E	Yes	No
193	B9H01_RS08590	*serS*	serine--tRNA ligase	J	Yes	Yes
194	B9H01_RS08665	*fabF*	beta-ketoacyl-ACP synthase II	I	Yes	No
195	B9H01_RS08675	*fabD*	ACP S-malonyltransferase	I	Yes	No
196	B9H01_RS08685	*acpP*	acyl carrier protein	IQ	No	Yes
197	B9H01_RS08690	*fabH*	beta-ketoacyl-ACP synthase III	I	Yes	No
198	B9H01_RS08725	-	alanine racemase	E	Yes	Yes
199	B9H01_RS08735	*secA*	preprotein translocase subunit SecA	U	No	Yes

No.	*S. suis* SC19 Locus tag	Name	Description	COG category	Metabolic model predicting essential genes	Tn-seq screening essential genes
200	B9H01_RS08790	-	magnesium transporter CorA family protein	P	Yes	Yes
201	B9H01_RS08805	*rpsR*	30S ribosomal protein S18	J	Yes	No
202	B9H01_RS08810	-	single-stranded DNA-binding protein	L	Yes	No
203	B9H01_RS08815	-	30S ribosomal protein S6	J	Yes	Yes
204	B9H01_RS08835	*lpdA*	dihydrolipoyl dehydrogenase	C	No	Yes
205	B9H01_RS08875	*infB*	translation initiation factor IF-2	J	No	Yes
206	B9H01_RS08890	*nusA*	transcription termination/antitermination protein NusA	K	Yes	Yes
207	B9H01_RS08950	-	DAK2 domain-containing protein	S	No	Yes
208	B9H01_RS09020	-	undecaprenyl/decaprenyl-phosphate alpha-N-acetylglucosaminyl 1-phosphate transferase	M	No	Yes
209	B9H01_RS09030	-	undecaprenyl-diphosphate phosphatase	V	Yes	No
210	B9H01_RS09055	*ilvA*	threonine ammonia-lyase IlvA	E	Yes	No
211	B9H01_RS09065	*ilvN*	acetolactate synthase small subunit	E	Yes	No
212	B9H01_RS09070	-	acetolactate synthase large subunit	E	Yes	No
213	B9H01_RS09090	*ilvD*	dihydroxy-acid dehydratase	E	Yes	No
214	B9H01_RS09110	*rpsI*	30S ribosomal protein S9	J	Yes	No

No.	*S. suis* SC19 Locus tag	Name	Description	COG category	Metabolic model predicting essential genes	Tn-seq screening essential genes
215	B9H01_RS09115	*rplM*	50S ribosomal protein L13	J	Yes	No
216	B9H01_RS09240	*cysS*	cysteine--tRNA ligase	J	Yes	Yes
217	B9H01_RS09300	*rpsO*	30S ribosomal protein S15	J	Yes	Yes
218	B9H01_RS09360	*leuB*	isopropylmalate dehydrogenase	CE	Yes	No
219	B9H01_RS09365	-	2-isopropylmalate synthase	E	Yes	No
220	B9H01_RS09390	-	PolC-type DNA polymerase III	L	No	Yes
221	B9H01_RS09425	-	proline--tRNA ligase	J	No	Yes
222	B9H01_RS09435	-	phosphatidate cytidylyltransferase	S	Yes	No
223	B9H01_RS09440	*uppS*	isoprenyl transferase	I	Yes	No
224	B9H01_RS09455	*gshAB*	bifunctional glutamate--cysteine ligase GshA/glutathione synthetase GshB	HM	Yes	No
225	B9H01_RS09480	*pnuC*	nicotinamide riboside transporter PnuC	NA	Yes	No
226	B9H01_RS09510	*tsf*	elongation factor Ts	J	No	Yes
227	B9H01_RS09515	*rpsB*	30S ribosomal protein S2	J	Yes	No
228	B9H01_RS09540	*secE*	preprotein translocase subunit SecE	U	No	Yes
229	B9H01_RS09685	*rpmH*	50S ribosomal protein L34	J	Yes	No
230	B9H01_RS09700	*rnpA*	ribonuclease P protein component	J	Yes	No
231	B9H01_RS09745	*gltX*	glutamate--tRNA ligase	J	Yes	Yes
232	B9H01_RS09810	*galU*	UTP--glucose-1-phosphate uridylyltransferase GalU	M	Yes	No

No.	*S. suis* SC19 Locus tag	Name	Description	COG category	Metabolic model predicting essential genes	Tn-seq screening essential genes
233	B9H01_RS09990	*leuS*	leucine--tRNA ligase	J	Yes	Yes
234	B9H01_RS10000	-	metal ABC transporter permease	P	Yes	Yes
235	B9H01_RS10005	-	iron chelate uptake ABC transporter family permease subunit	P	Yes	No
236	B9H01_RS10010	-	metal ABC transporter ATP-binding protein	P	Yes	No
237	B9H01_RS10230	*argS*	arginine--tRNA ligase	J	Yes	Yes
238	B9H01_RS10295	*aspS*	aspartate--tRNA ligase	J	Yes	Yes
239	B9H01_RS10355	*rpsD*	30S ribosomal protein S4	J	Yes	Yes
240	B9H01_RS10370	*dnaB*	replicative DNA helicase	L	No	Yes
241	B9H01_RS10375	-	50S ribosomal protein L9	J	Yes	No
242	B9H01_RS10435	-	energy-coupling factor transporter transmembrane protein EcfT	P	No	Yes
243	B9H01_RS10450	*pgsA*	CDP-diacylglycerol--glycerol-3-phosphate 3-phosphatidyltransferase	I	Yes	Yes
244	B9H01_RS10485	*trpS*	tryptophan--tRNA ligase	J	Yes	No

NA: not available

Table S3 Homology analysis of *S. suis* essential genes with other *Streptococcus* and *E. coli*

No.	***S. suis* SC19 Locus tag**	**Name**	**Description**	**Host homology**	***Streptococcus* genus core genome Locus tag**	***S. pneumoniae* D39 essential genes Locus tag**	***S. agalactiae* A909 essential genes Locus tag**	***S. equi* 4047 essential genes Locus tag**	***E. coli* K-12 essential genes Locus tag**
1	B9H01_RS00005	*dnaA*	chromosomal replication initiator protein DnaA	No	spr0001	SPD_0001	SAK_0001	SEQ_0001	b3702
2	B9H01_RS00010	*dnaN*	DNA polymerase III subunit beta	No	smi_0002	SPD_2045	SAK_0002	SEQ_0002	b3701
3	B9H01_RS00035	-	aminoacyl-tRNA hydrolase	No	smi_0005	SPD_0005	SAK_0007	NA	b1204
4	B9H01_RS00065	*tilS*	tRNA lysidine(34) synthetase TilS	No	spr0010	SPD_0011	SAK_0013	SEQ_0013	b0188
5	B9H01_RS00205	-	ribose-phosphate diphosphokinase	Yes	SPy_0020	SPD_0033	SAK_0051	SEQ_0020	b1207
6	B9H01_RS00440	*rpsJ*	30S ribosomal protein S10	No	spr0187	SPD_0192	SAK_0090	SEQ_0054	b3321

No.	*S. suis* SC19 Locus tag	Name	Description	Host homology	*Streptococcus* genus core genome Locus tag	*S. pneumoniae* D39 essential genes Locus tag	*S. agalactiae* A909 essential genes Locus tag	*S. equi* 4047 essential genes Locus tag	*E. coli* K-12 essential genes Locus tag
7	B9H01_RS00445	*rplC*	50S ribosomal protein L3	No	SAG0058	SPD_0193	SAK_0091	SEQ_0055	b3320
8	B9H01_RS00450	*rplD*	50S ribosomal protein L4	Yes	SPy_0050	SPD_0194	SAK_0092	SEQ_0056	b3319
9	B9H01_RS00455	*rplW*	50S ribosomal protein L23	No	smi_1903	SPD_0195	SAK_0093	SEQ_0057	b3318
10	B9H01_RS00460	*rplB*	50S ribosomal protein L2	No	spr0191	SPD_0196	SAK_0094	SEQ_0058	b3317
11	B9H01_RS00465	*rpsS*	30S ribosomal protein S19	Yes	smi_1901	SPD_0197	SAK_0095	SEQ_0059	b3316
12	B9H01_RS00470	*rplV*	50S ribosomal protein L22	No	spr0194	SPD_0198	SAK_0096	SEQ_0060	b3315
13	B9H01_RS00475	*rpsC*	30S ribosomal protein S3	No	SAG0064	SPD_0199	SAK_0097	SEQ_0061	b3314

No.	*S. suis* SC19 Locus tag	Name	Description	Host homology	*Streptococcus* genus core genome Locus tag	*S. pneumoniae* D39 essential genes Locus tag	*S. agalactiae* A909 essential genes Locus tag	*S. equi* 4047 essential genes Locus tag	*E. coli* K-12 essential genes Locus tag
14	B9H01_RS00480	*rplP*	50S ribosomal protein L16	No	smi_1898	SPD_0200	SAK_0098	SEQ_0062	b3313
15	B9H01_RS00485	*rpmC*	50S ribosomal protein L29	No	SSA_0115	SPD_0201	SAK_0099	SEQ_0063	b3312
16	B9H01_RS00490	*rpsQ*	30S ribosomal protein S17	No	SSA_0116	SPD_0202	SAK_0100	NA	b3311
17	B9H01_RS00495	*rplN*	50S ribosomal protein L14	No	SAG0068	SPD_0203	SAK_0101	SEQ_0065	b3310
18	B9H01_RS00500	*rplX*	50S ribosomal protein L24	No	smi_1894	SPD_0204	SAK_0102	NA	b3309
19	B9H01_RS00505	*rplE*	50S ribosomal protein L5	No	SSUBM407_0078	SPD_0205	SAK_0103	NA	b3308
20	B9H01_RS00510	-	type Z 30S ribosomal protein S14	No	NA	SPD_0206	SAK_0104	NA	b3307

No.	*S. suis* SC19 Locus tag	Name	Description	Host homology	*Streptococcus* genus core genome Locus tag	*S. pneumoniae* D39 essential genes Locus tag	*S. agalactiae* A909 essential genes Locus tag	*S. equi* 4047 essential genes Locus tag	*E. coli* K-12 essential genes Locus tag
21	B9H01_RS00520	*rpsH*	30S ribosomal protein S8	No	smi_1891	SPD_0207	SAK_0105	NA	b3306
22	B9H01_RS00530	*rplF*	50S ribosomal protein L6	No	SSA_0122	SPD_0208	SAK_0106	SEQ_0070	b3305
23	B9H01_RS00535	*rplR*	50S ribosomal protein L18	No	SSUBM407_0085	SPD_0209	NA	NA	b3304
24	B9H01_RS00540	*rpsE*	30S ribosomal protein S5	No	spr0206	SPD_0210	SAK_0108	SEQ_0072	b3303
25	B9H01_RS00545	*rpmD*	50S ribosomal protein L30	No	A2G56_RS06695	SPD_0211	SAK_0109	SEQ_0073	b3302
26	B9H01_RS00550	*rplO*	50S ribosomal protein L15	No	smi_1886	SPD_0212	SAK_0110	SEQ_0074	b3301
27	B9H01_RS00560	-	adenylate kinase	Yes	smi_1884	SPD_0214	SAK_0112	NA	b0474

No.	*S. suis* SC19 Locus tag	Name	Description	Host homology	*Streptococcus* genus core genome Locus tag	*S. pneumoniae* D39 essential genes Locus tag	*S. agalactiae* A909 essential genes Locus tag	*S. equi* 4047 essential genes Locus tag	*E. coli* K-12 essential genes Locus tag
28	B9H01_RS00570	*rpmJ*	50S ribosomal protein L36	Yes	NA	NA	NA	NA	NA
29	B9H01_RS00575	*rpsM*	30S ribosomal protein S13	No	spr0213	SPD_0216	SAK_0114	SEQ_0079	b3298
30	B9H01_RS00580	*rpsK*	30S ribosomal protein S11	Yes	spr0214	SPD_0217	SAK_0115	SEQ_0080	b3297
31	B9H01_RS00585	*rpoA*	DNA-directed RNA polymerase subunit alpha	No	smi_1879	SPD_0215	SAK_0116	SEQ_0081	b3295
32	B9H01_RS00590	*rplQ*	50S ribosomal protein L17	No	SSUBM407_0096	SPD_0219	SAK_0117	SEQ_0082	b3294
33	B9H01_RS00715	-	hypothetical protein	No	NA	NA	NA	NA	NA

No.	*S. suis* SC19 Locus tag	Name	Description	Host homology	*Streptococcus* genus core genome Locus tag	*S. pneumoniae* D39 essential genes Locus tag	*S. agalactiae* A909 essential genes Locus tag	*S. equi* 4047 essential genes Locus tag	*E. coli* K-12 essential genes Locus tag
34	B9H01_RS00770	*tyrS*	tyrosine--tRNA ligase	No	smi_0139	SPD_1926	SAK_0221	SEQ_0096	b1637
35	B9H01_RS00780	*rpoB*	DNA-directed RNA polymerase subunit beta	No	SPy_0098	SPD_1759	SAK_0223	SEQ_0098	b3987
36	B9H01_RS00785	*rpoC*	DNA-directed RNA polymerase subunit beta	No	spr1776	SPD_1758	SAK_0224	SEQ_0099	b3988
37	B9H01_RS00900	*groL*	chaperonin GroEL	Yes	smi_0482	SPD_1709	NA	NA	b4143
38	B9H01_RS00905	*rpsL*	30S ribosomal protein S12	Yes	smi_0261	SPD_0251	NA	NA	b3342
39	B9H01_RS00910	*rpsG*	30S ribosomal protein S7	No	smi_0262	SPD_0252	SAK_1792	SEQ_0341	b3341

No.	*S. suis* SC19 Locus tag	Name	Description	Host homology	*Streptococcus* genus core genome Locus tag	*S. pneumoniae* D39 essential genes Locus tag	*S. agalactiae* A909 essential genes Locus tag	*S. equi* 4047 essential genes Locus tag	*E. coli* K-12 essential genes Locus tag
40	B9H01_RS00915	*fusA*	elongation factor G	Yes	smi_0263	SPD_0253	SAK_1791	SEQ_0344	b3340
41	B9H01_RS00925	*gap*	type I glyceraldehyde-3-phosphate dehydrogenase	Yes	spr1825	SPD_1823	SAK_1790	SEQ_0345	b1779
42	B9H01_RS00970	*tsaD*	tRNA (adenosine(37)-N6)-threonylcarbamoyltransferase complex transferase subunit TsaD	No	spr0131	SPD_0136	SAK_1779	SEQ_0361	b3064
43	B9H01_RS01245	*treC*	alpha,alpha-phosphotrehalase	No	NA	SPD_1663	NA	NA	NA
44	B9H01_RS01280	*trxA*	thioredoxin	Yes	spr1602	SPD_1567	SAK_1725	SEQ_0401	NA

No.	*S. suis* SC19 Locus tag	Name	Description	Host homology	*Streptococcus* genus core genome Locus tag	*S. pneumoniae* D39 essential genes Locus tag	*S. agalactiae* A909 essential genes Locus tag	*S. equi* 4047 essential genes Locus tag	*E. coli* K-12 essential genes Locus tag
45	B9H01_RS01320	-	mechanosensitive ion channel family protein	No	SPy_1897	NA	NA	NA	NA
46	B9H01_RS01335	*gdhA*	NADP-specific glutamate dehydrogenase	No	NA	NA	NA	NA	NA
47	B9H01_RS01405	-	formate/nitrite transporter family protein	No	NA	NA	NA	NA	NA
48	B9H01_RS01450	*rpmF*	50S ribosomal protein L32	No	NA	NA	SAK_2048	NA	b1089
49	B9H01_RS01455	*hisS*	histidine--tRNA ligase	No	spr1931	SPD_1950	SAK_2047	SEQ_2174	b2514
50	B9H01_RS01505	*mvk*	mevalonate kinase	Yes	smi_1747	SPD_0346	SAK_1357	SEQ_1104	NA
51	B9H01_RS01510	*mvaD*	diphosphomevalonate decarboxylase	No	NA	SPD_0347	SAK_1356	SEQ_1105	NA

No.	*S. suis* SC19 Locus tag	Name	Description	Host homology	*Streptococcus* genus core genome Locus tag	*S. pneumoniae* D39 essential genes Locus tag	*S. agalactiae* A909 essential genes Locus tag	*S. equi* 4047 essential genes Locus tag	*E. coli* K-12 essential genes Locus tag
52	B9H01_RS01515	-	phosphomevalonate kinase type 2	No	NA	SPD_0348	SAK_1355	NA	NA
53	B9H01_RS01520	*fni*	isopentenyl-diphosphate Delta-isomerase	No	NA	SPD_2055	NA	SEQ_1107	NA
54	B9H01_RS01555	*grpE*	nucleotide exchange factor GrpE	No	spr0454	NA	SAK_0146	SEQ_0473	b2614
55	B9H01_RS01730	*rpmB*	50S ribosomal protein L28	No	NA	NA	NA	NA	b3637
56	B9H01_RS01810	*rplS*	50S ribosomal protein L19	No	smi_1265	SPD_1148	SAK_0692	NA	b2606
57	B9H01_RS01820	*gatC*	Asp-tRNA(Asn)/Glu-tRNA(Gln) amidotransferase subunit GatC	No	spr0395	SPD_0398	SAK_1681	SEQ_0461	NA

No.	*S. suis* SC19 Locus tag	Name	Description	Host homology	*Streptococcus* genus core genome Locus tag	*S. pneumoniae* D39 essential genes Locus tag	*S. agalactiae* A909 essential genes Locus tag	*S. equi* 4047 essential genes Locus tag	*E. coli* K-12 essential genes Locus tag
58	B9H01_RS01825	*gatA*	Asp-tRNA(Asn)/Glu-tRNA(Gln) amidotransferase subunit GatA	No	spr0394	SPD_0397	SAK_1680	SEQ_0462	NA
59	B9H01_RS01830	*gatB*	Asp-tRNA(Asn)/Glu-tRNA(Gln) amidotransferase subunit GatB	No	smi_1667	SPD_0396	SAK_1679	SEQ_0463	NA
60	B9H01_RS01930	-	nucleotidyltransferase	No	NA	NA	NA	NA	NA
61	B9H01_RS02070	*gpsB*	cell division regulator GpsB	No	spr0332	SPD_0339	NA	SEQ_0626	NA
62	B9H01_RS02100	*gmk*	guanylate kinase	Yes	smi_1628	SPD_1548	SAK_0383	SEQ_1780	b3648
63	B9H01_RS02105	-	DNA-directed RNA polymerase subunit omega	No	SAG0314	SPD_1547	NA	SEQ_1779	NA

No.	*S. suis* SC19 Locus tag	Name	Description	Host homology	*Streptococcus* genus core genome Locus tag	*S. pneumoniae* D39 essential genes Locus tag	*S. agalactiae* A909 essential genes Locus tag	*S. equi* 4047 essential genes Locus tag	*E. coli* K-12 essential genes Locus tag
64	B9H01_RS02110	-	primosomal protein N'	No	spr1581	SPD_1546	NA	SEQ_1778	NA
65	B9H01_RS02115	*fmt*	methionyl-tRNA formyltransferase	No	smi_1625	SPD_1545	SAK_0386	SEQ_1777	b3288
66	B9H01_RS02330	*valS*	valine--tRNA ligase	No	SMU_1770	SPD_0494	SAK_0547	SEQ_1672	b4258
67	B9H01_RS02365	*asnA*	aspartate--ammonia ligase	No	NA	NA	NA	NA	NA
68	B9H01_RS02420	*murG*	UDP-N-acetylglucosamine--N-acetylmuramyl-(pentapeptide) pyrophosphoryl-undecaprenol N-acetylglucosamine transferase	No	smi_1448	SPD_0599	SAK_0578	SEQ_0618	b0090

No.	*S. suis* SC19 Locus tag	Name	Description	Host homology	*Streptococcus* genus core genome Locus tag	*S. pneumoniae* D39 essential genes Locus tag	*S. agalactiae* A909 essential genes Locus tag	*S. equi* 4047 essential genes Locus tag	*E. coli* K-12 essential genes Locus tag
69	B9H01_RS02435	*ftsZ*	cell division protein FtsZ	No	spr1510	SPD_1479	SAK_0581	SEQ_0621	b0095
70	B9H01_RS02470	*ileS*	isoleucine--tRNA ligase	No	smi_0627	SPD_1472	SAK_0587	SEQ_0627	b0026
71	B9H01_RS02620	*lysS*	lysine--tRNA ligase	Yes	smi_1296	SPD_0620	SAK_0876	SEQ_1525	b2890
72	B9H01_RS02660	-	FtsW/RodA/SpoVE family cell cycle protein	No	spr0973	NA	NA	SEQ_0777	b0089
73	B9H01_RS02740	-	6-phosphofructokinase	No	smi_1004	SPD_0789	SAK_1036	SEQ_1011	NA
74	B9H01_RS02745	*pyk*	pyruvate kinase	No	smi_1005	SPD_0790	SAK_1037	SEQ_1012	NA
75	B9H01_RS02770	*glmS*	glutamine--fructose-6-phosphate transaminase (isomerizing)	No	spr0245	SPD_0248	SAK_1040	SEQ_1014	b3729

No.	*S. suis* SC19 Locus tag	Name	Description	Host homology	*Streptococcus* genus core genome Locus tag	*S. pneumoniae* D39 essential genes Locus tag	*S. agalactiae* A909 essential genes Locus tag	*S. equi* 4047 essential genes Locus tag	*E. coli* K-12 essential genes Locus tag
76	B9H01_RS02805	*proB*	glutamate 5-kinase	Yes	NA	NA	NA	NA	NA
77	B9H01_RS02810	-	glutamate-5-semialdehyde dehydrogenase	No	smi_0936	NA	NA	NA	NA
78	B9H01_RS02815	*proC*	pyrroline-5-carboxylate reductase	Yes	NA	NA	NA	NA	NA
79	B9H01_RS02925	*neuB*	N-acetylneuraminate synthase	No	NA	NA	NA	NA	NA
80	B9H01_RS02930	*neuC*	UDP-N-acetylglucosamine 2-epimerase (hydrolyzing)	No	NA	NA	NA	NA	NA
81	B9H01_RS02940	-	GDSL-type esterase/lipase family protein	No	NA	NA	NA	NA	b0918

No.	*S. suis* SC19 Locus tag	Name	Description	Host homology	*Streptococcus* genus core genome Locus tag	*S. pneumoniae* D39 essential genes Locus tag	*S. agalactiae* A909 essential genes Locus tag	*S. equi* 4047 essential genes Locus tag	*E. coli* K-12 essential genes Locus tag
82	B9H01_RS03070	*asnS*	asparagine--tRNA ligase	No	SSUBM407_1251	SPD_1371	SAK_0677	SEQ_0854	b0930
83	B9H01_RS03145	-	YfcC family protein	No	NA	NA	NA	NA	NA
84	B9H01_RS03200	*dltA*	D-alanine--poly(phosphoribitol) ligase subunit DltA	Yes	smi_2031	NA	NA	NA	NA
85	B9H01_RS03205	*dltB*	D-alanyl-lipoteichoic acid biosynthesis protein DltB	No	spr1981	NA	NA	NA	NA
86	B9H01_RS03215	*dltD*	D-alanyl-lipoteichoic acid biosynthesis protein DltD	No	spr1979	NA	NA	NA	NA

No.	*S. suis* SC19 Locus tag	Name	Description	Host homology	*Streptococcus* genus core genome Locus tag	*S. pneumoniae* D39 essential genes Locus tag	*S. agalactiae* A909 essential genes Locus tag	*S. equi* 4047 essential genes Locus tag	*E. coli* K-12 essential genes Locus tag
87	B9H01_RS03265	-	1-acyl-sn-glycerol-3-phosphate acyltransferase	No	spr1465	NA	SAK_0905	SEQ_0676	NA
88	B9H01_RS03300	*tmk*	dTMP kinase	No	smi_0938	SPD_0825	SAK_1591	SEQ_1829	b1098
89	B9H01_RS03305	-	DNA polymerase III subunit delta'	No	smi_0939	NA	SAK_1590	SEQ_1828	b1099
90	B9H01_RS03430	-	ECF transporter S component	No	smi_1195	NA	NA	NA	NA
91	B9H01_RS03440	-	phosphopantothenate--cysteine ligase	No	spr1110	SPD_1088	NA	NA	b3639
92	B9H01_RS03615	*pcrA*	DNA helicase PcrA	No	spr0995	SPD_0973	NA	SEQ_1021	NA
93	B9H01_RS03745	*parE*	DNA topoisomerase IV subunit B	No	smi_0897	SPD_0746	SAK_1243	SEQ_1340	b3699

No.	*S. suis* SC19 Locus tag	Name	Description	Host homology	*Streptococcus* genus core genome Locus tag	*S. pneumoniae* D39 essential genes Locus tag	*S. agalactiae* A909 essential genes Locus tag	*S. equi* 4047 essential genes Locus tag	*E. coli* K-12 essential genes Locus tag
94	B9H01_RS03780	-	branched-chain amino acid aminotransferase	No	spr0758	NA	NA	NA	NA
95	B9H01_RS03840	*rplU*	50S ribosomal protein L21	No	smi_0926	SPD_0989	SAK_1403	NA	b3186
96	B9H01_RS03845	*rpmA*	50S ribosomal protein L27	Yes	SSA_1062	SPD_0991	SAK_1401	NA	b3185
97	B9H01_RS03885	-	aspartate carbamoyltransferase catalytic subunit	No	smi_1235	NA	NA	NA	NA
98	B9H01_RS03915	*rpsP*	30S ribosomal protein S16	Yes	SSA_1310	SPD_0674	SAK_1391	SEQ_1308	b2609
99	B9H01_RS03920	-	KH domain-containing protein	No	spr0683	SPD_0675	SAK_1390	NA	NA

No.	*S. suis* SC19 Locus tag	Name	Description	Host homology	*Streptococcus* genus core genome Locus tag	*S. pneumoniae* D39 essential genes Locus tag	*S. agalactiae* A909 essential genes Locus tag	*S. equi* 4047 essential genes Locus tag	*E. coli* K-12 essential genes Locus tag
100	B9H01_RS03950	*murB*	UDP-N-acetylmuramate dehydrogenase	No	spr1247	NA	SAK_1197	SEQ_1128	b3972
101	B9H01_RS04060	*ccA*	CCA tRNA nucleotidyltransferase	No	smi_1546	SPD_1386	SAK_1372	SEQ_1286	b3056
102	B9H01_RS04090	-	thymidylate synthase	No	smi_1477	SPD_0581	SAK_1346	NA	b2827
103	B9H01_RS04110	-	ribosome biogenesis GTP-binding protein YihA/YsxC	Yes	smi_1558	SPD_1398	SAK_1342	SEQ_1114	b3865
104	B9H01_RS04150	*prfA*	peptide chain release factor 1	Yes	smi_1127	SPD_0906	SAK_1163	SEQ_1161	b1211
105	B9H01_RS04770	-	transcriptional regulator	No	NA	SPD_1565	NA	NA	NA

No.	*S. suis* SC19 Locus tag	Name	Description	Host homology	*Streptococcus* genus core genome Locus tag	*S. pneumoniae* D39 essential genes Locus tag	*S. agalactiae* A909 essential genes Locus tag	*S. equi* 4047 essential genes Locus tag	*E. coli* K-12 essential genes Locus tag
106	B9H01_RS04855	*rplJ*	50S ribosomal protein L10	No	spr1212	SPD_1188	SAK_1335	SEQ_1391	b3985
107	B9H01_RS04860	*topA*	type I DNA topoisomerase	No	spr1141	SPD_1120	NA	SEQ_1219	b4566
108	B9H01_RS04910	*ylqF*	ribosome biogenesis GTPase YlqF	Yes	NA	SPD_1019	SAK_1108	SEQ_1222	b2511
109	B9H01_RS05190	*nadK*	NAD kinase	No	spr1005	SPD_0983	SAK_1179	SEQ_1144	b2615
110	B9H01_RS05245	*gyrA*	DNA gyrase subunit A	No	spr1099	SPD_1077	SAK_1055	SEQ_1170	b2231
111	B9H01_RS05285	*deoC*	deoxyribose-phosphate aldolase	No	smi_0890	NA	NA	NA	NA
112	B9H01_RS05300	-	type I pantothenate kinase	No	spr0741	SPD_0733	NA	SEQ_1056	b3974

No.	*S. suis* SC19 Locus tag	Name	Description	Host homology	*Streptococcus* genus core genome Locus tag	*S. pneumoniae* D39 essential genes Locus tag	*S. agalactiae* A909 essential genes Locus tag	*S. equi* 4047 essential genes Locus tag	*E. coli* K-12 essential genes Locus tag
113	B9H01_RS05305	-	30S ribosomal protein S20	No	SPy_1234	NA	NA	NA	b0023
114	B9H01_RS05375	*pstS*	phosphate ABC transporter substrate-binding protein PstS family protein	No	spr1257	NA	SAK_1087	NA	NA
115	B9H01_RS05405	*ribC*	bifunctional riboflavin kinase/FAD synthetase	No	spr1017	SPD_0994	SAK_1092	SEQ_1037	b0025
116	B9H01_RS05585	*pheT*	phenylalanine--tRNA ligase subunit beta	No	smi_1801	SPD_0483	SAK_0994	SEQ_0930	b1713
117	B9H01_RS05595	*pheS*	phenylalanine--tRNA ligase subunit alpha	No	smi_1803	SPD_0504	SAK_0992	SEQ_0929	b1714
118	B9H01_RS05685	*atpG*	F0F1 ATP synthase subunit gamma	No	spr1361	SPD_1336	SAK_0985	SEQ_0920	NA

No.	*S. suis* SC19 Locus tag	Name	Description	Host homology	*Streptococcus* genus core genome Locus tag	*S. pneumoniae* D39 essential genes Locus tag	*S. agalactiae* A909 essential genes Locus tag	*S. equi* 4047 essential genes Locus tag	*E. coli* K-12 essential genes Locus tag
119	B9H01_RS05690	-	F0F1 ATP synthase subunit alpha	Yes	spr1362	SPD_1337	SAK_0984	SEQ_0919	NA
120	B9H01_RS05710	-	F0F1 ATP synthase subunit C	No	NA	SPD_1341	SAK_0980	NA	NA
121	B9H01_RS05765	*smc*	chromosome segregation protein SMC	Yes	spr1126	SPD_1104	NA	NA	NA
122	B9H01_RS05770	*rnc*	ribonuclease III	Yes	smi_1210	SPD_1105	SAK_0849	SEQ_1567	NA
123	B9H01_RS05800	*ptsP*	phosphoenolpyruvate--protein phosphotransferase	No	spr1062	SPD_1039	SAK_0946	SEQ_0890	b2416
124	B9H01_RS05805	*ptsH*	phosphocarrier protein HPr	No	smi_1045	SPD_1040	SAK_0945	SEQ_0889	NA
125	B9H01_RS05825	*nrdH*	glutaredoxin-like protein NrdH	No	spr1064	NA	SAK_0944	SEQ_0888	NA

No.	*S. suis* SC19 Locus tag	Name	Description	Host homology	*Streptococcus* genus core genome Locus tag	*S. pneumoniae* D39 essential genes Locus tag	*S. agalactiae* A909 essential genes Locus tag	*S. equi* 4047 essential genes Locus tag	*E. coli* K-12 essential genes Locus tag
126	B9H01_RS05830	*nrdE*	class 1b ribonucleoside-diphosphate reductase subunit alpha	No	spr1065	SPD_1042	SAK_0943	SEQ_0887	b2234
127	B9H01_RS05835	-	hypothetical protein	No	NA	NA	NA	NA	NA
128	B9H01_RS05960	-	helix-turn-helix transcriptional regulator	No	NA	NA	SAK_0235	NA	NA
129	B9H01_RS05985	*alaS*	alanine--tRNA ligase	No	spr1240	SPD_1216	SAK_0934	SEQ_0695	b2697
130	B9H01_RS06015	*pepF*	oligoendopeptidase F	No	smi_0981	NA	NA	NA	NA
131	B9H01_RS06025	*metG*	methionine--tRNA ligase	No	spr0696	SPD_0689	SAK_1995	SEQ_1672	b2114

No.	*S. suis* SC19 Locus tag	Name	Description	Host homology	*Streptococcus* genus core genome Locus tag	*S. pneumoniae* D39 essential genes Locus tag	*S. agalactiae* A909 essential genes Locus tag	*S. equi* 4047 essential genes Locus tag	*E. coli* K-12 essential genes Locus tag
132	B9H01_RS06135	*rplT*	50S ribosomal protein L20	No	SAG1382	NA	SAK_1415	NA	b1716
133	B9H01_RS06140	*rpmI*	50S ribosomal protein L35	No	NA	SPD_0848	SAK_1416	SEQ_0982	b1717
134	B9H01_RS06145	*infC*	translation initiation factor IF-3	No	spr0861	SPD_0847	SAK_1417	SEQ_0981	b1718
135	B9H01_RS06260	*rmlD*	dTDP-4-dehydrorhamnose reductase	No	NA	SPD_0331	SAK_1459	SEQ_0962	NA
136	B9H01_RS06280	*rmlA*	glucose-1-phosphate thymidylyltransferase RfbA	No	NA	SPD_0328	SAK_1287	SEQ_1419	NA
137	B9H01_RS06345	*rnz*	ribonuclease Z	No	smi_1472	SPD_0586	SAK_1296	SEQ_1429	NA
138	B9H01_RS06410	*pyrH*	UMP kinase	No	spr0845	SPD_0834	SAK_1537	SEQ_1643	b0171

**No.**	***S. suis* SC19 Locus tag**	**Name**	**Description**	**Host homology**	***Streptococcus* genus core genome Locus tag**	***S. pneumoniae* D39 essential genes Locus tag**	***S. agalactiae* A909 essential genes Locus tag**	***S. equi* 4047 essential genes Locus tag**	***E. coli* K-12 essential genes Locus tag**
139	B9H01_RS06415	-	polysaccharide biosynthesis protein	No	spr1383	SPD_1358	SAK_1423	SEQ_1834	NA
140	B9H01_RS06430	*rplA*	50S ribosomal protein L1	No	spr0556	NA	SAK_1543	SEQ_1650	b3984
141	B9H01_RS06435	*rplK*	50S ribosomal protein L11	No	SAG1520	SPD_0550	SAK_1544	NA	b3983
142	B9H01_RS06530	*ddl*	D-alanine--D-alanine ligase	No	smi_0615	SPD_1484	SAK_0892	SEQ_0662	NA
143	B9H01_RS06560	*vicK*	cell wall metabolism sensor histidine kinase VicK	No	spr1106	SPD_1084	NA	NA	NA
144	B9H01_RS06565	*vicR*	response regulator transcription factor	No	SPy_0528	SPD_1085	SAK_0845	SEQ_1577	NA

No.	*S. suis* SC19 Locus tag	Name	Description	Host homology	*Streptococcus* genus core genome Locus tag	*S. pneumoniae* D39 essential genes Locus tag	*S. agalactiae* A909 essential genes Locus tag	*S. equi* 4047 essential genes Locus tag	*E. coli* K-12 essential genes Locus tag
145	B9H01_RS06590	*thrS*	threonine--tRNA ligase	No	spr1472	SPD_1444	SAK_0837	SEQ_1591	b1719
146	B9H01_RS06650	*polA*	DNA polymerase I	No	spr0032	SPD_0038	NA	SEQ_0252	NA
147	B9H01_RS06745	-	diacylglycerol kinase family protein	No	spr0870	SPD_0856	NA	SEQ_1634	NA
148	B9H01_RS06815	-	ABC transporter permease	No	spr0667	SPD_1290	NA	SEQ_0784	NA
149	B9H01_RS06825	*prfB*	peptide chain release factor 2	No	spr0665	SPD_0658	SAK_0668	SEQ_0782	b2891
150	B9H01_RS06905	*rpoD*	RNA polymerase sigma factor RpoD	No	smi_0992	SPD_0958	SAK_1461	SEQ_0960	b3067
151	B9H01_RS06910	*dnaG*	DNA primase	No	spr0978	SPD_0957	SAK_1462	SEQ_0959	b3066

No.	*S. suis* SC19 Locus tag	Name	Description	Host homology	*Streptococcus* genus core genome Locus tag	*S. pneumoniae* D39 essential genes Locus tag	*S. agalactiae* A909 essential genes Locus tag	*S. equi* 4047 essential genes Locus tag	*E. coli* K-12 essential genes Locus tag
152	B9H01_RS06920	*galE*	UDP-glucose 4-epimerase GalE	Yes	NA	SPD_1432	SAK_1882	SEQ_1417	NA
153	B9H01_RS06925	*rpsU*	30S ribosomal protein S21	No	NA	SPD_1245	SAK_1464	SEQ_0956	b3065
154	B9H01_RS06955	-	VanZ family protein	No	NA	NA	NA	NA	NA
155	B9H01_RS06975	-	arsenate reductase	No	NA	NA	NA	NA	NA
156	B9H01_RS06980	-	phosphopentomutase	No	spr0732	NA	NA	NA	NA
157	B9H01_RS07095	*cdaA*	TIGR00159 family protein	No	spr1419	SPD_1392	SAK_1008	SEQ_1399	NA
158	B9H01_RS07190	-	beta-glucoside-specific PTS transporter subunit IIABC	No	NA	NA	NA	NA	NA

No.	*S. suis* SC19 Locus tag	Name	Description	Host homology	*Streptococcus* genus core genome Locus tag	*S. pneumoniae* D39 essential genes Locus tag	*S. agalactiae* A909 essential genes Locus tag	*S. equi* 4047 essential genes Locus tag	*E. coli* K-12 essential genes Locus tag
159	B9H01_RS07285	*gyrB*	DNA topoisomerase (ATP-hydrolyzing) subunit B	No	smi_0860	SPD_0709	SAK_0708	SEQ_0894	b3699
160	B9H01_RS07390	*rpmE*	type B 50S ribosomal protein L31	No	SSUBM407_1425	SPD_1154	SAK_0686	SEQ_0874	NA
161	B9H01_RS07440	*holA*	DNA polymerase III subunit delta	No	spr0673	SPD_0666	SAK_0912	SEQ_0679	NA
162	B9H01_RS07535	-	cystathionine gamma-synthase	Yes	NA	NA	NA	NA	NA
163	B9H01_RS07835	-	methionine adenosyltransferase	Yes	spr0671	SPD_0664	SAK_0954	SEQ_0706	b2942
164	B9H01_RS07865	*dnaX*	DNA polymerase III subunit gamma/tau	No	spr0769	SPD_0757	SAK_0952	SEQ_0703	b0470

No.	*S. suis* SC19 Locus tag	Name	Description	Host homology	*Streptococcus* genus core genome Locus tag	*S. pneumoniae* D39 essential genes Locus tag	*S. agalactiae* A909 essential genes Locus tag	*S. equi* 4047 essential genes Locus tag	*E. coli* K-12 essential genes Locus tag
165	B9H01_RS07920	-	acetyl-CoA C-acetyltransferase	Yes	NA	NA	NA	NA	NA
166	B9H01_RS07925	*mvaS*	hydroxymethyl glutaryl-CoA synthase	No	spr1571	SPD_1537	SAK_1347	SEQ_1109	NA
167	B9H01_RS07930	*mvaA*	hydroxymethyl glutaryl-CoA reductase,degradative	No	smi_1618	SPD_1522	SAK_1348	SEQ_1108	NA
168	B9H01_RS07955	-	HU family DNA-binding protein	No	SSA_0683	NA	SAK_0606	NA	b1712
169	B9H01_RS07990	-	farnesyl diphosphate synthase	No	smi_1016	NA	NA	NA	b0421
170	B9H01_RS08075	-	nicotinate phosphoribosyl transferase	No	spr1277	NA	SAK_0367	SEQ_1793	NA
171	B9H01_RS08180	-	ECF transporter S component	No	spr0433	NA	NA	SEQ_1849	NA

No.	*S. suis* SC19 Locus tag	Name	Description	Host homology	*Streptococcus* genus core genome Locus tag	*S. pneumoniae* D39 essential genes Locus tag	*S. agalactiae* A909 essential genes Locus tag	*S. equi* 4047 essential genes Locus tag	*E. coli* K-12 essential genes Locus tag
172	B9H01_RS08205	*scpB*	SMC-Scp complex subunit ScpB	No	smi_0338	SPD_1655	NA	NA	NA
173	B9H01_RS08210	-	segregation/condensation protein A	No	spr1691	NA	NA	NA	NA
174	B9H01_RS08235	*murI*	glutamate racemase	No	spr1696	SPD_1661	SAK_1615	SEQ_1868	b3967
175	B9H01_RS08245	-	diaminopimelate decarboxylase	No	NA	NA	NA	NA	NA
176	B9H01_RS08275	*yidC*	membrane protein insertase YidC	No	smi_0310	SPD_1773	NA	SEQ_1878	b3705
177	B9H01_RS08295	*murC*	UDP-N-acetylmuramate--L-alanine ligase	No	spr1373	SPD_1349	SAK_1630	SEQ_1882	b0091
178	B9H01_RS08335	*dnaI*	primosomal protein DnaI	No	smi_0584	SPD_1521	SAK_1635	SEQ_1886	NA

No.	*S. suis* SC19 Locus tag	Name	Description	Host homology	*Streptococcus* genus core genome Locus tag	*S. pneumoniae* D39 essential genes Locus tag	*S. agalactiae* A909 essential genes Locus tag	*S. equi* 4047 essential genes Locus tag	*E. coli* K-12 essential genes Locus tag
179	B9H01_RS08340	-	replication initiation/membrane attachment protein	No	smi_0583	SPD_1522	SAK_1636	SEQ_1887	NA
180	B9H01_RS08360	*gndA*	NADP-dependent phosphogluconate dehydrogenase	Yes	NA	NA	NA	NA	NA
181	B9H01_RS08380	*deaD*	DEAD/DEAH box helicase	No	spr0670	SPD_0659	SAK_0361	SEQ_1801	NA
182	B9H01_RS08390	*mraY*	phospho-N-acetylmuramoyl-pentapeptide-transferase	No	spr0305	SPD_0307	SAK_0360	SEQ_1802	b0087
183	B9H01_RS08395	*pbp2X*	penicillin-binding protein PBP2X	No	spr0304	SPD_0306	SAK_0359	SEQ_1803	b0084
184	B9H01_RS08460	-	polyprenyl synthetase family protein	No	NA	NA	NA	NA	b3187

No.	*S. suis* SC19 Locus tag	Name	Description	Host homology	*Streptococcus* genus core genome Locus tag	*S. pneumoniae* D39 essential genes Locus tag	*S. agalactiae* A909 essential genes Locus tag	*S. equi* 4047 essential genes Locus tag	*E. coli* K-12 essential genes Locus tag
185	B9H01_RS08470	-	Gx transporter family protein	No	NA	NA	NA	NA	NA
186	B9H01_RS08480	-	potassium transporter Trk	No	NA	NA	NA	SEQ_1895	NA
187	B9H01_RS08485	-	TrkA family potassium uptake protein	No	NA	NA	NA	SEQ_1896	NA
188	B9H01_RS08505	*glyS*	glycine--tRNA ligase subunit beta	No	smi_1395	SPD_1285	SAK_0342	SEQ_0544	b3559
189	B9H01_RS08510	*glyQ*	glycine--tRNA ligase subunit alpha	No	smi_1396	SPD_1305	SAK_0340	SEQ_0543	b3560
190	B9H01_RS08525	-	methionine ABC transporter permease	No	spr0150	NA	NA	NA	NA
191	B9H01_RS08540	-	MetQ/NlpA family ABC transporter substrate-binding protein	No	spr0147	NA	NA	NA	NA

No.	*S. suis* SC19 Locus tag	Name	Description	Host homology	*Streptococcus* genus core genome Locus tag	*S. pneumoniae* D39 essential genes Locus tag	*S. agalactiae* A909 essential genes Locus tag	*S. equi* 4047 essential genes Locus tag	*E. coli* K-12 essential genes Locus tag
192	B9H01_RS08555	*metF*	methylenetetrahydrofolate reductase [NAD(P)H]	No	NA	NA	NA	NA	NA
193	B9H01_RS08590	*serS*	serine--tRNA ligase	No	spr0372	SPD_0375	SAK_0837	SEQ_1591	b0893
194	B9H01_RS08665	*fabF*	beta-ketoacyl-ACP synthase II	No	SPy_1748	SPD_0385	SAK_0423	SEQ_0483	b2323
195	B9H01_RS08675	*fabD*	ACP S-malonyltransferase	No	spr0380	SPD_0383	SAK_0421	SEQ_0481	b1092
196	B9H01_RS08685	*acpP*	acyl carrier protein	No	spr0378	SPD_0381	SAK_0419	SEQ_0479	b1094
197	B9H01_RS08690	*fabH*	beta-ketoacyl-ACP synthase III	No	smi_1684	SPD_0380	SAK_0418	SEQ_0478	b1091

No.	*S. suis* SC19 Locus tag	Name	Description	Host homology	*Streptococcus* genus core genome Locus tag	*S. pneumoniae* D39 essential genes Locus tag	*S. agalactiae* A909 essential genes Locus tag	*S. equi* 4047 essential genes Locus tag	*E. coli* K-12 essential genes Locus tag
198	B9H01_RS08725	-	alanine racemase	No	spr1540	SPD_1508	SAK_1696	NA	NA
199	B9H01_RS08735	*secA*	preprotein translocase subunit SecA	No	spr1544	SPD_1512	NA	SEQ_0435	b0098
200	B9H01_RS08790	-	magnesium transporter CorA family protein	No	spr0170	SPD_0175	SAK_1719	SEQ_0415	NA
201	B9H01_RS08805	*rpsR*	30S ribosomal protein S18	No	SSA_0440	SPD_1368	SAK_1720	NA	b4202
202	B9H01_RS08810	-	single-stranded DNA-binding protein	No	smi_1522	SPD_1369	SAK_1721	NA	b4059
203	B9H01_RS08815	-	30S ribosomal protein S6	No	smi_1523	SPD_1370	NA	NA	b4200
204	B9H01_RS08835	*lpdA*	dihydrolipoyl dehydrogenase	No	spr1048	NA	SAK_0366	SEQ_1403	b0116

No.	*S. suis* SC19 Locus tag	Name	Description	Host homology	*Streptococcus* genus core genome Locus tag	*S. pneumoniae* D39 essential genes Locus tag	*S. agalactiae* A909 essential genes Locus tag	*S. equi* 4047 essential genes Locus tag	*E. coli* K-12 essential genes Locus tag
205	B9H01_RS08875	*infB*	translation initiation factor IF-2	Yes	smi_0379	SPD_0482	SAK_0455	SEQ_0518	b3168
206	B9H01_RS08890	*nusA*	transcription termination/antitermination protein NusA	No	smi_0376	SPD_0479	NA	SEQ_0515	b3169
207	B9H01_RS08950	-	DAK2 domain-containing protein	No	spr0400	SPD_0403	SAK_0182	SEQ_1938	NA
208	B9H01_RS09020	-	undecaprenyl/decaprenyl-phosphate alpha-N-acetylglucosaminyl 1-phosphate transferase	No	NA	NA	SAK_0198	SEQ_1931	b0087
209	B9H01_RS09030	-	undecaprenyl-diphosphate phosphatase	No	smi_1817	SPD_0402	NA	NA	NA

No.	*S. suis* SC19 Locus tag	Name	Description	Host homology	*Streptococcus* genus core genome Locus tag	*S. pneumoniae* D39 essential genes Locus tag	*S. agalactiae* A909 essential genes Locus tag	*S. equi* 4047 essential genes Locus tag	*E. coli* K-12 essential genes Locus tag
210	B9H01_RS09055	*ilvA*	threonine ammonia-lyase IlvA	No	NA	NA	NA	NA	NA
211	B9H01_RS09065	*ilvN*	acetolactate synthase small subunit	No	NA	NA	NA	NA	NA
212	B9H01_RS09070	-	acetolactate synthase large subunit	No	NA	SPD_0636	NA	NA	NA
213	B9H01_RS09090	*ilvD*	dihydroxy-acid dehydratase	No	NA	NA	NA	NA	NA
214	B9H01_RS09110	*rpsI*	30S ribosomal protein S9	Yes	spr0272	SPD_0275	SAK_0277	NA	b3230
215	B9H01_RS09115	*rplM*	50S ribosomal protein L13	No	SPy_1932	SPD_0274	SAK_0276	NA	b3231
216	B9H01_RS09240	*cysS*	cysteine--tRNA ligase	No	smi_1595	SPD_0515	SAK_0270	SEQ_1992	b0526

No.	*S. suis* SC19 Locus tag	Name	Description	Host homology	*Streptococcus* genus core genome Locus tag	*S. pneumoniae* D39 essential genes Locus tag	*S. agalactiae* A909 essential genes Locus tag	*S. equi* 4047 essential genes Locus tag	*E. coli* K-12 essential genes Locus tag
217	B9H01_RS09300	*rpsO*	30S ribosomal protein S15	No	SPy_1955	SPD_1439	SAK_0265	NA	b3165
218	B9H01_RS09360	*leuB*	isopropylmalate dehydrogenase	No	NA	NA	NA	NA	NA
219	B9H01_RS09365	-	2-isopropylmalate synthase	No	NA	NA	NA	NA	NA
220	B9H01_RS09390	-	PolC-type DNA polymerase III	No	smi_0264	NA	SAK_1869	SEQ_2003	b0184
221	B9H01_RS09425	-	proline--tRNA ligase	Yes	spr0243	SPD_0246	SAK_1871	SEQ_2005	b0194
222	B9H01_RS09435	-	phosphatidate cytidylyltransferase	No	smi_1854	SPD_0244	SAK_1873	SEQ_2007	b0175
223	B9H01_RS09440	*uppS*	isoprenyl transferase	No	spr0240	SPD_0243	SAK_1874	SEQ_2008	b0174

No.	*S. suis* SC19 Locus tag	Name	Description	Host homology	*Streptococcus* genus core genome Locus tag	*S. pneumoniae* D39 essential genes Locus tag	*S. agalactiae* A909 essential genes Locus tag	*S. equi* 4047 essential genes Locus tag	*E. coli* K-12 essential genes Locus tag
224	B9H01_RS09455	*gshAB*	bifunctional glutamate--cysteine ligase GshA/glutathione synthetase GshB	No	NA	NA	NA	NA	NA
225	B9H01_RS09480	*pnuC*	nicotinamide riboside transporter PnuC	No	NA	NA	NA	NA	NA
226	B9H01_RS09510	*tsf*	elongation factor Ts	No	spr2019	SPD_2041	SAK_1851	SEQ_0129	b0170
227	B9H01_RS09515	*rpsB*	30S ribosomal protein S2	No	spr2020	SPD_2042	NA	SEQ_0130	b0169
228	B9H01_RS09540	*secE*	preprotein translocase subunit SecE	No	NA	SPD_1820	SAK_2003	NA	NA
229	B9H01_RS09685	*rpmH*	50S ribosomal protein L34	No	NA	SPD_1790	SAK_1815	NA	b3703

No.	*S. suis* SC19 Locus tag	Name	Description	Host homology	*Streptococcus* genus core genome Locus tag	*S. pneumoniae* D39 essential genes Locus tag	*S. agalactiae* A909 essential genes Locus tag	*S. equi* 4047 essential genes Locus tag	*E. coli* K-12 essential genes Locus tag
230	B9H01_RS09700	*rnpA*	ribonuclease P protein component	No	smi_0215	SPD_1851	SAK_0481	SEQ_0304	b3704
231	B9H01_RS09745	*gltX*	glutamate--tRNA ligase	No	SPy_0239	SPD_1896	SAK_0165	SEQ_0300	b2400
232	B9H01_RS09810	*galU*	UTP--glucose-1-phosphate uridylyltransferase GalU	No	spr1903	SPD_1919	SAK_0479	SEQ_1419	NA
233	B9H01_RS09990	*leuS*	leucine--tRNA ligase	No	SAG2057	SPD_0238	SAK_1995	SEQ_0234	NA
234	B9H01_RS10000	-	metal ABC transporter permease	No	NA	NA	NA	SEQ_1658	NA
235	B9H01_RS10005	-	iron chelate uptake ABC transporter family permease subunit	No	NA	NA	NA	SEQ_1658	NA

No.	*S. suis* SC19 Locus tag	Name	Description	Host homology	*Streptococcus* genus core genome Locus tag	*S. pneumoniae* D39 essential genes Locus tag	*S. agalactiae* A909 essential genes Locus tag	*S. equi* 4047 essential genes Locus tag	*E. coli* K-12 essential genes Locus tag
236	B9H01_RS10010	-	metal ABC transporter ATP-binding protein	No	smi_0636	SPD_1450	SAK_0364	SEQ_0552	b1117
237	B9H01_RS10230	*argS*	arginine--tRNA ligase	No	smi_0150	SPD_1905	SAK_2042	SEQ_2164	b1876
238	B9H01_RS10295	*aspS*	aspartate--tRNA ligase	Yes	spr1924	SPD_1941	SAK_2046	SEQ_2169	b1866
239	B9H01_RS10355	*rpsD*	30S ribosomal protein S4	No	SSUBM407_2000	SPD_0083	SAK_2095	SEQ_2201	b3296
240	B9H01_RS10370	*dnaB*	replicative DNA helicase	No	SPy_2182	SPD_2030	SAK_2097	SEQ_2203	b4052
241	B9H01_RS10375	-	50S ribosomal protein L9	No	smi_2054	NA	NA	SEQ_2204	NA
242	B9H01_RS10435	-	energy-coupling factor transporter transmembrane protein EcfT	No	spr2024	NA	SAK_2107	SEQ_2213	NA

No.	*S. suis* SC19 Locus tag	Name	Description	Host homology	*Streptococcus* genus core genome Locus tag	*S. pneumoniae* D39 essential genes Locus tag	*S. agalactiae* A909 essential genes Locus tag	*S. equi* 4047 essential genes Locus tag	*E. coli* K-12 essential genes Locus tag
243	B9H01_RS10450	*pgsA*	CDP-diacylglycerol--glycerol-3-phosphate 3-phosphatidyltransferase	No	spr2027	NA	SAK_2110	NA	b1912
244	B9H01_RS10485	*trpS*	tryptophan--tRNA ligase	No	spr2034	SPD_2056	NA	SEQ_2231	b3384

NA: not available

Table S4 Potential anti-streptococcal drug targets

No.	*S. suis* SC19 Locus tag	Name	Description	COG category	COG category description
1	**B9H01_RS00005**	***dnaA***	chromosomal replication initiator protein DnaA	L	Replication, recombination and repair
2	**B9H01_RS00010**	***dnaN***	DNA polymerase III subunit beta	L	Replication, recombination and repair
3	**B9H01_RS00065**	***tilS***	tRNA lysidine(34) synthetase TilS	J	Translation, ribosomal structure and biogenesis
4	**B9H01_RS00440**	***rpsJ***	30S ribosomal protein S10	J	Translation, ribosomal structure and biogenesis
5	**B9H01_RS00445**	***rplC***	50S ribosomal protein L3	J	Translation, ribosomal structure and biogenesis
6	**B9H01_RS00455**	***rplW***	50S ribosomal protein L23	J	Translation, ribosomal structure and biogenesis
7	**B9H01_RS00460**	***rplB***	50S ribosomal protein L2	J	Translation, ribosomal structure and biogenesis
8	**B9H01_RS00470**	***rplV***	50S ribosomal protein L22	J	Translation, ribosomal structure and biogenesis
9	**B9H01_RS00475**	***rpsC***	30S ribosomal protein S3	J	Translation, ribosomal structure and biogenesis
10	**B9H01_RS00480**	***rplP***	50S ribosomal protein L16	J	Translation, ribosomal structure and biogenesis
11	**B9H01_RS00485**	***rpmC***	50S ribosomal protein L29	J	Translation, ribosomal structure and biogenesis
12	**B9H01_RS00495**	***rplN***	50S ribosomal protein L14	J	Translation, ribosomal structure and biogenesis
13	**B9H01_RS00530**	***rplF***	50S ribosomal protein L6	J	Translation, ribosomal structure and biogenesis
14	**B9H01_RS00540**	***rpsE***	30S ribosomal protein S5	J	Translation, ribosomal structure and biogenesis
15	**B9H01_RS00545**	***rpmD***	50S ribosomal protein L30	J	Translation, ribosomal structure and biogenesis
16	**B9H01_RS00550**	***rplO***	50S ribosomal protein L15	J	Translation, ribosomal structure and biogenesis
17	**B9H01_RS00575**	***rpsM***	30S ribosomal protein S13	J	Translation, ribosomal structure and biogenesis
18	**B9H01_RS00585**	***rpoA***	DNA-directed RNA polymerase subunit alpha	K	Transcription
19	**B9H01_RS00590**	***rplQ***	50S ribosomal protein L17	J	Translation, ribosomal structure and biogenesis
20	**B9H01_RS00770**	***tyrS***	tyrosine--tRNA ligase	J	Translation, ribosomal structure and biogenesis
21	**B9H01_RS00780**	***rpoB***	DNA-directed RNA polymerase subunit beta	K	Transcription
22	**B9H01_RS00785**	***rpoC***	DNA-directed RNA polymerase subunit beta	K	Transcription
23	**B9H01_RS00910**	***rpsG***	30S ribosomal protein S7	J	Translation, ribosomal structure and biogenesis

No.	*S. suis* SC19 Locus tag	Name	Description	COG category	COG category description
24	**B9H01_RS00970**	***tsaD***	tRNA (adenosine(37)-N6)-threonylcarbamoyltransferase complex transferase subunit TsaD	O	Posttranslational modification, protein turnover, chaperones
25	**B9H01_RS01455**	***hisS***	histidine--tRNA ligase	J	Translation, ribosomal structure and biogenesis
26	B9H01_RS01510	*mvaD*	diphosphomevalonate decarboxylase	I	Lipid transport and metabolism
27	B9H01_RS01820	*gatC*	Asp-tRNA(Asn)/Glu-tRNA(Gln) amidotransferase subunit GatC	J	Translation, ribosomal structure and biogenesis
28	B9H01_RS01825	*gatA*	Asp-tRNA(Asn)/Glu-tRNA(Gln) amidotransferase subunit GatA	J	Translation, ribosomal structure and biogenesis
29	B9H01_RS01830	*gatB*	Asp-tRNA(Asn)/Glu-tRNA(Gln) amidotransferase subunit GatB	J	Translation, ribosomal structure and biogenesis
30	**B9H01_RS02115**	***fmt***	methionyl-tRNA formyltransferase	J	Translation, ribosomal structure and biogenesis
31	**B9H01_RS02330**	***valS***	valine--tRNA ligase	J	Translation, ribosomal structure and biogenesis
32	**B9H01_RS02420**	***murG***	UDP-N-acetylglucosamine--N-acetylmuramyl-(pentapeptide) pyrophosphoryl-undecaprenol N-acetylglucosamine transferase	M	Cell wall/membrane/envelope biogenesis
33	**B9H01_RS02435**	***ftsZ***	cell division protein FtsZ	D	Cell cycle control, cell division, chromosome partitioning
34	**B9H01_RS02470**	***ileS***	isoleucine--tRNA ligase	J	Translation, ribosomal structure and biogenesis
35	B9H01_RS02745	*pyk*	pyruvate kinase	G	Carbohydrate transport and metabolism
36	**B9H01_RS02770**	***glmS***	glutamine--fructose-6-phosphate transaminase (isomerizing)	M	Cell wall/membrane/envelope biogenesis
37	**B9H01_RS03070**	***asnS***	asparagine--tRNA ligase	J	Translation, ribosomal structure and biogenesis
38	**B9H01_RS03300**	***tmk***	dTMP kinase	F	Nucleotide transport and metabolism
39	**B9H01_RS03745**	***parE***	DNA topoisomerase IV subunit B	L	Replication, recombination and repair

No.	*S. suis* SC19 Locus tag	Name	Description	COG category	COG category description
40	**B9H01_RS04060**	***ccA***	CCA tRNA nucleotidyltransferase	J	Translation, ribosomal structure and biogenesis
41	**B9H01_RS04855**	***rplJ***	50S ribosomal protein L10	J	Translation, ribosomal structure and biogenesis
42	**B9H01_RS05190**	***nadK***	NAD kinase	G	Carbohydrate transport and metabolism
43	**B9H01_RS05245**	***gyrA***	DNA gyrase subunit A	L	Replication, recombination and repair
44	**B9H01_RS05405**	***ribC***	bifunctional riboflavin kinase/FAD synthetase	H	Coenzyme transport and metabolism
45	**B9H01_RS05585**	***pheT***	phenylalanine--tRNA ligase subunit beta	J	Translation, ribosomal structure and biogenesis
46	**B9H01_RS05595**	***pheS***	phenylalanine--tRNA ligase subunit alpha	J	Translation, ribosomal structure and biogenesis
47	B9H01_RS05685	*atpG*	F0F1 ATP synthase subunit gamma	C	Energy production and conversion
48	**B9H01_RS05800**	***ptsP***	phosphoenolpyruvate--protein phosphotransferase	G	Carbohydrate transport and metabolism
49	B9H01_RS05805	*ptsH*	phosphocarrier protein HPr	G	Carbohydrate transport and metabolism
50	**B9H01_RS05830**	***nrdE***	class 1b ribonucleoside-diphosphate reductase subunit alpha	F	Nucleotide transport and metabolism
51	**B9H01_RS05985**	***alaS***	alanine--tRNA ligase	J	Translation, ribosomal structure and biogenesis
52	**B9H01_RS06025**	***metG***	methionine--tRNA ligase	J	Translation, ribosomal structure and biogenesis
53	**B9H01_RS06140**	***rpmI***	50S ribosomal protein L35	J	Translation, ribosomal structure and biogenesis
54	**B9H01_RS06145**	***infC***	translation initiation factor IF-3	J	Translation, ribosomal structure and biogenesis
55	B9H01_RS06260	*rmlD*	dTDP-4-dehydrorhamnose reductase	M	Cell wall/membrane/envelope biogenesis
56	B9H01_RS06280	*rmlA*	glucose-1-phosphate thymidylyltransferase RfbA	M	Cell wall/membrane/envelope biogenesis
57	B9H01_RS06345	*rnz*	ribonuclease Z	S	Function unknown
58	**B9H01_RS06410**	***pyrH***	UMP kinase	F	Nucleotide transport and metabolism

No.	*S. suis* SC19 Locus tag	Name	Description	COG category	COG category description
59	B9H01_RS06415	B9H01_RS06415	polysaccharide biosynthesis protein	S	Function unknown
60	B9H01_RS06530	*ddl*	D-alanine--D-alanine ligase	M	Cell wall/membrane/envelope biogenesis
61	B9H01_RS06565	*vicR*	response regulator transcription factor	T	Signal transduction mechanisms
62	**B9H01_RS06590**	***thrS***	threonine--tRNA ligase	J	Translation, ribosomal structure and biogenesis
63	**B9H01_RS06825**	***prfB***	peptide chain release factor 2	J	Translation, ribosomal structure and biogenesis
64	**B9H01_RS06905**	***rpoD***	RNA polymerase sigma factor RpoD	K	Transcription
65	**B9H01_RS06910**	***dnaG***	DNA primase	L	Replication, recombination and repair
66	**B9H01_RS06925**	***rpsU***	30S ribosomal protein S21	J	Translation, ribosomal structure and biogenesis
67	B9H01_RS07095	*cdaA*	TIGR00159 family protein	S	Function unknown
68	**B9H01_RS07285**	***gyrB***	DNA topoisomerase (ATP-hydrolyzing) subunit B	L	Replication, recombination and repair
69	B9H01_RS07390	*rpmE*	type B 50S ribosomal protein L31	J	Translation, ribosomal structure and biogenesis
70	B9H01_RS07440	*holA*	DNA polymerase III subunit delta	L	Replication, recombination and repair
71	**B9H01_RS07865**	***dnaX***	DNA polymerase III subunit gamma/tau	L	Replication, recombination and repair
72	B9H01_RS07925	*mvaS*	hydroxymethylglutaryl-CoA synthase	I	Lipid transport and metabolism
73	B9H01_RS07930	*mvaA*	hydroxymethylglutaryl-CoA reductase,degradative	I	Lipid transport and metabolism
74	**B9H01_RS08235**	***murI***	glutamate racemase	M	Cell wall/membrane/envelope biogenesis
75	**B9H01_RS08295**	***murC***	UDP-N-acetylmuramate--L-alanine ligase	M	Cell wall/membrane/envelope biogenesis
76	B9H01_RS08335	*dnaI*	primosomal protein DnaI	L	Replication, recombination and repair
77	B9H01_RS08340	B9H01_RS08340	replication initiation/membrane attachment protein	L	Replication, recombination and repair

No.	*S. suis* SC19 Locus tag	Name	Description	COG category	COG category description
78	B9H01_RS08380	*deaD*	DEAD/DEAH box helicase	L	Replication, recombination and repair
79	**B9H01_RS08390**	***mraY***	phospho-N-acetylmuramoyl-pentapeptide-transferase	M	Cell wall/membrane/envelope biogenesis
80	**B9H01_RS08395**	***pbp2X***	penicillin-binding protein PBP2X	M	Cell wall/membrane/envelope biogenesis
81	**B9H01_RS08505**	***glyS***	glycine--tRNA ligase subunit beta	J	Translation, ribosomal structure and biogenesis
82	**B9H01_RS08510**	***glyQ***	glycine--tRNA ligase subunit alpha	J	Translation, ribosomal structure and biogenesis
83	**B9H01_RS08590**	***serS***	serine--tRNA ligase	J	Translation, ribosomal structure and biogenesis
84	**B9H01_RS08665**	***fabF***	beta-ketoacyl-ACP synthase II	I	Lipid transport and metabolism
85	**B9H01_RS08675**	***fabD***	ACP S-malonyltransferase	I	Lipid transport and metabolism
86	**B9H01_RS08685**	***acpP***	acyl carrier protein	IQ	Lipid transport and metabolism
87	**B9H01_RS08690**	***fabH***	beta-ketoacyl-ACP synthase III	I	Lipid transport and metabolism
88	B9H01_RS08790	B9H01_RS08790	magnesium transporter CorA family protein	P	Inorganic ion transport and metabolism
89	B9H01_RS08950	B9H01_RS08950	DAK2 domain-containing protein	S	Function unknown
90	**B9H01_RS09240**	***cysS***	cysteine--tRNA ligase	J	Translation, ribosomal structure and biogenesis
91	**B9H01_RS09435**	**B9H01_RS09435**	phosphatidate cytidylyltransferase	S	Function unknown
92	**B9H01_RS09440**	***uppS***	isoprenyl transferase	I	Lipid transport and metabolism
93	**B9H01_RS09510**	***tsf***	elongation factor Ts	J	Translation, ribosomal structure and biogenesis
94	**B9H01_RS09700**	***rnpA***	ribonuclease P protein component	J	Translation, ribosomal structure and biogenesis

No.	*S. suis* SC19 Locus tag	Name	Description	COG category	COG category description
95	**B9H01_RS09745**	***gltX***	glutamate--tRNA ligase	J	Translation, ribosomal structure and biogenesis
96	B9H01_RS09810	*galU*	UTP--glucose-1-phosphate uridylyltransferase GalU	M	Cell wall/membrane/envelope biogenesis
97	B9H01_RS09990	*leuS*	leucine--tRNA ligase	J	Translation, ribosomal structure and biogenesis
98	**B9H01_RS10010**	**B9H01_RS10010**	metal ABC transporter ATP-binding protein	P	Inorganic ion transport and metabolism
99	**B9H01_RS10230**	***argS***	arginine--tRNA ligase	J	Translation, ribosomal structure and biogenesis
100	**B9H01_RS10355**	***rpsD***	30S ribosomal protein S4	J	Translation, ribosomal structure and biogenesis
101	**B9H01_RS10370**	***dnaB***	replicative DNA helicase	L	Replication, recombination and repair

Broad spectrum antibacterial drug targets are represented in bold font (Locus tag and Name).
